# Soil properties drive nitrous oxide accumulation patterns by shaping denitrifying bacteriomes

**DOI:** 10.1186/s40793-024-00643-9

**Published:** 2024-11-21

**Authors:** Saira Bano, Qiaoyu Wu, Siyu Yu, Xinhui Wang, Xiaojun Zhang

**Affiliations:** State Key Laboratory of Microbial metabolism, Joint International Research Laboratory of Metabolic & Developmental Sciences, School of Life Sciences & Biotechnology, Shanghai Jiao Tong University, 800 Dongchuan Road, Minhang District, Shanghai, 200240 China

**Keywords:** Soil properties, Nitrous oxide, Denitrification, Denitrifying bacteria, Denitrifying genes

## Abstract

In agroecosystems, nitrous oxide (N₂O) emissions are influenced by both microbiome composition and soil properties, yet the relative importance of these factors in determining differential N₂O emissions remains unclear. This study investigates the impacts of these factors on N₂O emissions using two primary agricultural soils from northern China: fluvo-aquic soil (FS) from the North China Plain and black soil (BS) from Northeast China, which exhibit significant differences in physicochemical properties. In non-sterilized controls (NSC), we observed distinct denitrifying bacterial phenotypes between FS and BS, with BS exhibiting significantly higher N₂O emissions. Cross-inoculation experiments were conducted by introducing extracted microbiomes into sterile recipient soils of both types to disentangle the relative contributions of soil properties and microbiomes on N₂O emission potential. The results showed recipient-soil-dependent gas kinetics, with significantly higher N₂O/(N₂O + N₂) ratios in BS compared to FS, regardless of the inoculum type. Metagenomic analysis further revealed significant shifts in denitrification genes and microbial diversity of the inoculated bacteriomes influenced by the recipient soil. The higher ratios of *nirS/nosZ* in FS and *nirK/nosZ* in BS indicated that the recipient soil dictates the formation of different denitrifying guilds. Specifically, the BS environment fosters *nirK*-based denitrifiers like *Rhodanobacter*, contributing to higher N₂O accumulation, while FS supports a diverse array of denitrifiers, including *Pseudomonas* and *Stutzerimonas*, associated with complete denitrification and lower N₂O emissions. This study underscores the critical role of soil properties in shaping microbial community dynamics and greenhouse gas emissions. These findings highlight the importance of considering soil physicochemical properties in managing agricultural practices to mitigate N₂O emissions.

**Supplementary Information:**

The online version contains supplementary material available at 10.1186/s40793-024-00643-9.

## Introduction

Nitrous oxide (N₂O) is a long-lived stratospheric ozone-depleting potent greenhouse gas with a current atmospheric lifetime of 116 ± 9 years [1]. According to the latest IPCC assessment report, over a 100-year time scale, the global warming potential of N₂O is estimated to be 273 times greater than that of CO₂ [2]. Agroecosystems are significant contributors globally, representing 60% of anthropogenic global N₂O emissions [3], primarily produced from microbially mediated processes such as denitrification [4].

Denitrification is a microbially mediated process that typically occurs under anoxic conditions when nitrate (NO_3_⁻) or nitrite (NO_2_⁻) is used as a terminal electron acceptor and reduced to nitrogen (N) gases (dinitrogen (N_2_) and N_2_O) [5]. Complete denitrification is the stepwise reduction of NO_3_⁻ to N_2_, catalyzed by a series of complex metalloenzymes encoded by genes such as *narG* (NO_3_⁻ reductase), *nirK* (copper-containing NO_2_⁻ reductase), *nirS* (cytochrome cd-containing NO_2_⁻ reductase), *norB* (nitric oxide (NO) reductase), and *nosZ* (N_2_O reductase) [6]. True denitrifiers inherit a complete set of denitrification functional genes and are considered key players in the process [7]. However, the process is usually affected by the diversity and abundance of the denitrifying microbial community [8, 9], as organisms lacking one or more of these genes are called truncated or incomplete denitrifiers and can contribute to significant N_2_O emissions [6, 7].

To date, in agroecosystems, studies have often explained N_2_O fluxes based on either the abundance and diversity of denitrifying microbial communities [9, 10] or variations in soil physicochemical properties [3, 7, 11–15]. Despite advancements in understanding the proximal (microbial) and distal (soil properties) controls influencing N₂O emissions, the relative contributions of soil properties and microbial community dynamics remain unclear. Soil types, with their distinct physicochemical characteristics such as pH [12], moisture content [13], and nutrient availability [3, 7] can play a pivotal role in shaping microbial community structure and function [16, 17]. For example, when pH value was lower than 6 the activity of nitrous oxide reductase enzyme would be ultimately constrained, resulting in more N_2_O emissions [12]. Similarly, fertilizer-induced changes in soil pH and organic matter attributes reshape distinct denitrifying guilds and ultimately affect N_2_O emissions [18, 19].

According to FAOSTAT (2014), China, despite having only 7% of the world’s arable land, produces enough food to feed 19% of the global population [20]. To achieve this, chemical fertilizers are heavily used to boost grain yields, often applied at levels exceeding the optimal requirements [7]. The North China Plain and Northeast China are key agricultural regions, known for large-scale wheat and corn cultivation, respectively [21, 22]. Overfertilization is a prevalent issue in these areas, with conventional practices applying 550 to 600 kg of N per hectare, far above recommended levels [23], which has led to significant NOx emissions [24] and making the region a major contributor to global N_2_O emissions [1]. In Northeast China, soils are characterized by low pH and are rich in organic matter, such as black soil, whereas the North China Plain is dominated by alkaline, calcareous fluvo-aquic soils that are low in carbon content [21, 25]. These soil types are markedly different in physicochemical properties and also the soil microbiomes [9], which likely affect the denitrification potential and N₂O emission rates of these soils differently. This underscores the necessity to dissect the interplay between soil properties and microbial community dynamics to develop effective strategies for mitigating N₂O emissions.

Effective management of N₂O emissions from agricultural soils requires an integrated approach that accounts for both microbial community composition and soil physicochemical properties [26, 27]. This study aims to elucidate the interactions between soil properties and microbial community dynamics, emphasizing their functional outcomes regarding N₂O emissions. Molecular approaches, particularly metagenomic analyses, offer a comprehensive method for tracking shifts in microbial abundance and diversity. They also enable the identification of key functional genes and taxa involved in denitrification processes, providing insights into the mechanisms that drive N₂O production and reduction in soils [28, 29].

Our hypothesis posits that soil properties act as distal drivers by shaping the selection of microbial communities—the proximal drivers of N₂O emissions—within the soil environment. This determinism suggests that specific soil environments favour the selection and persistence of microbiomes with particular functional capabilities, reflecting adaptation to the unique conditions of each soil. To test this hypothesis, we conducted extracted-microbiome-based cross-inoculation experiments between fluvo-aquic soil (FS) and black soil (BS). Sterile soils were inoculated with extracted microbiomes originating from either their native soil type or from the other soil type. Using particle-free microbial cell-extraction technology, we prepared microbiome inoculums by separating microbial cells from soil particles. We examined the patterns of N₂O emission kinetics across the treatments and analysed shifts in the structure of the total bacterial and denitrifying bacterial communities, as well as the relative and absolute abundances of key denitrifying genes. This experimental design enables us to disentangle the contributions of soil properties and inoculated microbiomes to the denitrifying bacterial communities and their N₂O emission potential. Understanding these dynamics is essential for developing targeted strategies to mitigate N₂O emissions in agricultural systems.

## Materials and methods

### Soil sample collection and preparation

Two distinct types of soil samples, FS from the North China Plain and BS from Northeast China, were collected from cropland in long-term experimental fields (Table S1), as detailed by Wu et al. [9]. For each soil type, samples were taken from five different points at depths of 0 to 20 cm and then mixed to create a composite sample. The soil samples were sieved through a 2 mm mesh and placed in black plastic bags. Before incubation experiments, a portion of each soil type was sterilized using gamma irradiation (36 kGy) and stored in closed plastic bags. All samples were kept at 4 °C until use.

Soil pH, NO_3_^-^-N, NO_2_^-^-N, ammonium (NH_4_^+^), and soil moisture content were measured as described previously [9]. Water holding capacity (WHC) was determined using the funnel method, following the procedure outlined in Govindasamy et al. [30] with some modifications (see supplementary material for details). The soils were preconditioned in the dark at 50% WHC and 25 °C for 7 days before the incubation experiment.

### Microbial cell extraction from the soil

The microbial cell extraction method was adapted from previous literatures [31, 32] with some modifications. Briefly, 20 g (dry weight) of soil sample for each replicate was taken separately in a 150 mL conical flask, pre-activated for one week at 25 °C, and made into 1:5 slurries with sterile water. Each technical replicate was vortexed for 1 min and then aliquoted into a Waring laboratory blender, where it was blended at full speed for five 1-minute intervals with intermittent cooling on ice. The dispersed soil suspensions were set aside for 30 min to allow the coarse soil particles to settle.

A density gradient was created by transferring the soil suspensions into centrifuge tubes and carefully placing an 80% nycodenz (Nycomed, Oslo, Norway) solution underneath the soil suspension in a 3:1 volume ratio (soil suspension: nycodenz). The tubes were then centrifuged in a high-speed swing-out rotor centrifuge at 4 °C and 14,000×g for 60 min. The middle layer of cells was carefully collected along with some nycodenz solution, excluding the pellet, and transferred into new tubes. These were diluted with sterile water and centrifuged at 14,000×g for 45 min. After discarding the supernatant, the pellet was resuspended in an appropriate amount of sterile water to adjust the WHC of the 20 g of sterilized recipient soil to 50%. The suspension was stored at 4 °C before inoculation. The extracted cells were counted based on the method described by Highton et al. [33].

### Experimental design for the microbial inoculation and anoxic incubation

Two types of soil samples, FS and BS, were arranged into three treatment groups: NSC (pre-activated non-sterilized control), FSB (sterilized soil inoculated with microbial inoculum extracted from pre-activated fluvo-aquic soil), and BSB (sterilized soil inoculated with microbial inoculum extracted from pre-activated black soil) (Fig. S1). Each treatment group included triplicate samples of 20 g (dry weight) of the respective soil, placed in 120 mL serum vials, with 50% WHC and adjusted to initial NO₃⁻ (KNO_3_) and carbon (C₆H₁₂O₆) contents of 250 mg/kg and 1000 mg/kg of soil, respectively.

To compare gas kinetics between the cell extracts and the parent soils, sterile sister soils (gamma-sterilized parent soils) were used for inoculating the cell extracts. This approach was necessary because the cell suspension alone could not fully replicate the soil matrix’s effects, particularly regarding gas diffusion and microbial niches. For each replicate, a particle-free cell extract extracted from 20 g of soil was inoculated into 20 g of sterile soil, which served as the extracted cell group for comparison with the 20 g of parent soil during gas kinetics measurement.

The sample vials were sealed with airtight butyl-rubber septa and aluminum crimp caps. Each replicate was inoculated with microbial cells extracted from 20 g (dry weight) of the respective soil, injected into the sealed vials via syringe through the rubber septa. Negative controls of sterile soils (without inoculation) were also prepared to confirm the sterility of the soils. The headspace of the serum vials was alternately evacuated and refilled with high-purity helium (99.999%) four times to create a completely anoxic environment. All vials were incubated at 25 °C, and the headspace was sampled every 6 h for N_2_O and N_2_ concentration analysis by an auto-sampler robotized incubation system (ROBOT), as described by Molstad et al. [34]. Triplicate vials were destructively sampled on days 0, 1, and 14 for molecular analysis.

The auto-sampler ROBOT system, equipped with a gas chromatograph (GC) (7890 A, Agilent, USA), was used to measure the kinetics of gaseous N. Briefly, a peristaltic pump transferred the headspace gas from the incubation vials to the GC system, where N_2_O and N_2_ concentrations were determined using an electron capture detector and a thermal conductivity detector, respectively. Besides measuring the N_2_O and N_2_ accumulation during the anoxic incubation, the N_2_O index (*I*_N₂O_) was also calculated to characterize the N_2_O production ratio expressed as N_2_O/(N_2_O + N_2_) as described previously [12]. *I*_N₂O_ formula is:

$$\:{I}_{{\text{N}}_{2}\text{O}}=\:{\int\:}_{0}^{{t}_{end}}{\text{N}}_{2}\text{O}(t)\text{d}t/{\int\:}_{0}^{{t}_{end}}\left[{\text{N}}_{2}\text{O}\left(t\right)+{\text{N}}_{2}\left(t\right)\right]\text{d}t$$

where N_2_O(*t*) represents the accumulation of N_2_O at any time *t*, N_2_(*t*) represents the accumulation of N_2_ at any time *t*, and *t*_*end*_ is the end time of the incubation.

### Soil DNA extraction and bioinformatics used for the community analysis

DNA was extracted from 0.3 g of frozen soil samples for each replicate using the FastDNA SPIN Kit (MP Biomedicals, USA), following the manufacturer’s instructions. A total of 54 DNA samples were collected from six treatment groups (triplicates per group) at three different time points (day 0, day 1, and day 14). The universal primers 341F and 785R were used to amplify the V3-V4 region of the 16S rRNA gene amplicons according to the Illumina MiSeq platform protocol (Illumina Inc., USA). PCR amplification was performed in triplicate for each DNA sample, and the purified amplified products were sequenced using an Illumina MiSeq system as described previously [9, 35].

The 16S rRNA gene sequence data was imported and pre-processed in QIIME [36] to check the quality and quantity of sequences and remove adapter sequences. The adapter-trimmed sequences were denoised, and the forward and reverse sequence reads were spliced together using DADA2 [37]. Specifically, the forward and reverse reads were truncated at positions 275 and 175, respectively, while discarding reads shorter than these values. A minimum 20-nucleotide overlap between the forward and reverse reads was required. Untrusted or chimeric sequences were eliminated, and taxonomy was assigned using the SILVA 138 database. A phylogenetic tree was constructed using FastTree [38]. The amplicon sequence variants (ASV) table was rarefied to a depth of 11,000 sequences per sample with 5,000 permutations before computing diversity metrics.

### Quantitation of nitrogen cycling genes

The quantity of extracted DNA was measured using the PicoGreen dsDNA Assay Kit (Quanti-iT PicoGreen, Invitrogen, USA) on a multi-mode microplate reader (SpectraMax iD5, USA) and used as the template. The copy numbers of bacterial denitrification-related genes were determined by quantitative PCR (qPCR) using a fluorescence quantitative PCR instrument (qTOWER3G touch, Analytik Jena, Germany). The primers and PCR conditions used for amplification were as described previously [9, 10]. Specifically, the bacterial *narG*, *nirS*, *nirK*, and *nosZ* genes were amplified using the primers narG-f/narG-r, nirS-cd3aF/nirS-R3cd, nirK-1040/F1aCu, and nosZ-2f/2r, respectively (Table S2).

### Metagenome sequencing and data analysis

To investigate the microbiome’s ecological functions across different treatment groups, soil samples from day 0 and day 14 were selected for metagenomic sequencing. The whole-genome shotgun (WGS) approach was employed to sequence total metagenomic DNA from the soil samples using the Illumina Novaseq high-throughput sequencing platform (Shanghai Majorbio Bio-pharm Technology Co., Ltd.). The sequencing was conducted with 2 × 150 bp paired-end reads, following the construction of a sequencing library from fragmented soil DNA.

Unless specified otherwise, all software used in this study utilized default parameters. Quality control of the raw sequencing data was performed using Fastp (v0.20.0) [39], resulting in a clean dataset for subsequent analysis. Contigs ≥ 300 bp were retained after the clean reads were assembled into contigs using MEGAHIT (v1.1.2) [40]. Coding sequences within the contigs were predicted using Prodigal (v2.6.3) [41]. The contigs were then clustered with 95% similarity and 90% alignment coverage to generate a non-redundant contig set using linclust mode of MMseqs2 [42]. Species annotation of the non-redundant gene sets was conducted by aligning them to the NCBI non-redundant (NR) database using BLASTP (v2.2.28+) with the DIAMOND (v2.0.13) [43] software program, employing the best-hit species annotation method. For functional gene annotation, the non-redundant gene set sequences were aligned to the Kyoto Encyclopedia of Genes and Genomes (KEGG) database using DIAMOND [43], and the predicted genes were annotated using KOBAS (v2.0) [44]. Gene abundance profiles were calculated using transcripts per million (TPM), which corresponds to mapped reads in this study. The TPM values were used to normalize the effects of total read counts and gene lengths, facilitating the comparison of gene abundances between samples [45].

### Statistical analysis

The fold change of ASV abundance differences between soils and extracted cells for the initial 16S rRNA gene V3-V4 region sequences was generated using the edgeR package [46] with an exact test. Taxonomy was assigned to ASVs only if their abundance differed by more than fivefold with a *p* < 1 × 10^− 5^ in the exact test output.

The statistical significance of the gas kinetics, the quantity of denitrifying genes, and the relative abundance of denitrifying genes based on metagenomic data was determined by a two-way analysis of variance (ANOVA) with a post hoc Tukey’s multiple comparisons test. Furthermore, significant differences in overall bacterial alpha diversity, denitrifying bacterial community alpha diversity indices, and the relative abundance of denitrifying bacterial genera between FS and BS across different treatment groups were determined using the Kruskal-Wallis statistical test. Additionally, Principal Coordinates Analysis (PCoA) based on Bray-Curtis distance was performed to construct ordination plots and visualize shifts in overall bacterial community composition and denitrifying bacterial communities across different treatments. These dissimilarity patterns were confirmed by the Analysis of Similarities (ANOSIM) test using the vegan package [47].

Furthermore, biomarker denitrifying species were identified using Linear Discriminant Analysis Effect Size (LEfSe) with parameters of *p* < 0.05 and LDA score > 2 [48]. Spearman’s correlation between LEfSe-derived key denitrifying species and N_2_O accumulation was performed in MATLAB 2022a.

The relationship between soil type, denitrifying communities, and the N_2_O index was explored using partial least squares path modeling (PLSPM). In the PLSPM model, the estimates of the coefficients of determination (R^2^) and the path coefficient (direct effect) were validated using the plspm package [49] with 1000 bootstraps.

Finally, all data were graphed using GraphPad Prism version 8.0 and R version 4.3.1 [50].

## Results

### Physicochemical properties of the two soils

The physicochemical properties of FS and BS displayed significant differences, as detailed in Table 1. Specifically, BS exhibited markedly higher values for moisture content, WHC, NO_3_⁻, and dissolved organic carbon (DOC) content compared to FS. Conversely, BS demonstrated a significantly lower NH_4_^+^ content. Additionally, the pH values differed substantially between the two samples: BS presented an acidic pH of 6.5, whereas FS showed an alkaline pH of 8.2.

**Table 1:** Differences in physicochemical properties of the two soils, displayed as mean values with SD from triplicate samples per treatment

	FS	BS	Sig
Moisture content %	2.3 ± 0.04	14.8 ± 0.08	***
WHC %	45.1 ± 0.47	48.7 ± 0.40	**
pH	8.2 ± 0.02	6.5 ± 0.01	***
NO_3_^−^ (mg/Kg soil)	1.2 ± 0.17	10.9 ± 3.41	**
NO_2_^−^ (mg/Kg soil)	0	0	–
NH_4_^+^ (mg/Kg soil)	10.9 ± 0.39	7.5 ± 0.93	**
DOC (mg/kg)	18.08 ± 0.96	26.95 ± 1.11	**

Sig = Significance. ** *p* ≤ 0.01; *** *p* ≤ 0.001

### Microbial cell extraction bias

To evaluate the representativeness of extracted cells to soil bacteriomes, 16S rRNA gene amplicon sequencing was employed. The ASVs predominantly correlated with their soil origin, as evidenced by the clustering of extracted cells near their original soils on the PCoA plot, distinct from other soils (ANOSIM: *R* = 0.97, *p* = 0.002, Fig. S2a). This analysis also revealed inter-soil differences. Non-significant changes were detected between extracted cells and their origin soils, both shared a substantial proportion of ASVs (mean 56.5%, standard deviation 10.8%) (Fig. S2b). Further, ASV detection bias was evident at the phylum level (Fig. S2c), with Acidobacteria being more prevalent in soils and Firmicutes in extracts. Differential abundance analysis using an exact test identified specific organisms with varying relative extractability (Fig. S2d). ASVs linked to Pyrinomonadaceae (Acidobacteria) and Bacillaceae (Firmicutes) were significantly less and more abundant in extracted cells compared to soils, respectively, with no other consistent extraction biases observed.

To further investigate the functional relatedness of extracted cells in comparison to soil bacteriomes, we analyzed the differences in N_2_O accumulation dynamics between original soils and their corresponding cell extracts. For FS (Fig. S3a), the cell extract accumulated relatively less N_2_O compared to the soil, although the overall trend of gas peaking and declining remained consistent between the two. In contrast, for BS (Fig. S3b), the cell extract exhibited a similar N_2_O accumulation to that of the soil, with a slightly quicker onset of accumulation. These findings demonstrate a functional similarity, indicating that the N_2_O accumulation profiles between cell extracts and their corresponding soils did not differ significantly.

### Comparison of gas kinetics during anoxic incubation

**Fig. 1:**
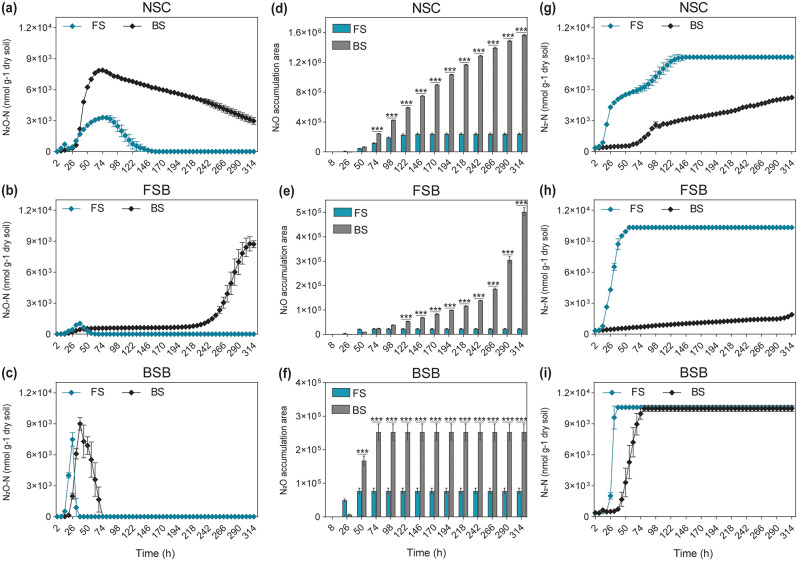
Dynamics of gas kinetics during anoxic incubation. Comparison of N_2_O **(a–c)** and N_2_**(g–i)** kinetics, and N_2_O accumulation area **(d–f)** between FS and BS across three different treatment groups: NSC, FSB, and BSB. Bars indicate means, and error bars represent the standard error of the mean (SEM). Significant differences are calculated via two-way ANOVA followed by Tukey’s multiple comparisons test and are indicated by asterisks (* *p* < 0.05, ** *p* < 0.01, *** *p* < 0.001)

For the N_2_O kinetics, in the NSC group (Fig. 1a), N₂O accumulation was significantly higher in the BS compared to FS throughout the incubation. Peak N₂O concentrations in BS reached approximately 8 × 10^3^ nmol g⁻¹ dry soil around 74 h, while FS maintained lower levels, peaking at around 4 × 10³ nmol g⁻¹ dry soil and completely depleted until 150 h while continued to decrease gradually in BS. The FSB treatment (Fig. 1b) showed a dramatic increase in N₂O accumulation in BS from 242 h onwards, reaching nearly 9 × 10^3^ nmol g⁻¹ dry soil by 314 h. In contrast, FS exhibited lower N₂O levels that completely declined before 74 h. The BSB group (Fig. 1c) on the other hand, demonstrated that BS had a notable increase in N₂O levels, peaking above 9 × 10^3^ nmol g⁻¹ dry soil around 38 h and gradually started declining afterwards. Whereas, FS treatment in the BSB group also showed an increase but peaked earlier at around 7 × 10³ nmol g⁻¹ dry soil by 26 h, followed by a sharp decline.

In the NSC group (Fig. 1d), accumulation area under the dynamic curve of N_2_O was significantly (*p* < 0.001) greater in BS compared to FS at all time points after 50 h. The FSB group (Fig. 1e) also exhibited significantly (*p* < 0.001) higher N₂O accumulation in BS than FS, especially notable after 98 h. The total N_2_O accumulation area of BS was 6.50 and 21.74 times higher than that of FS in both groups respectively. In the BSB group (Fig. 1f), accumulation area under the dynamic curve of N₂O was consistently higher in BS compared to FS, with significant differences (*p* < 0.001) observed from 50 h onwards and the total accumulation area of BS was 3.26 times greater than that of FS.

The N_2_ accumulation by FS was much higher than BS in all groups. In the NSC group (Fig. 1g), N_2_ accumulation was consistently higher in FS compared to BS throughout the incubation, peaking above 9 × 10^3^ nmol g⁻¹ dry soil. For the FSB group (Fig. 1h), FS exhibited a rapid increase in N₂ levels, stabilizing above 9 × 10^3^ nmol g⁻¹ dry soil before 50 h, while BS showed a super delayed and minimal accumulation. In the BSB group (Fig. 1i), compared to BS, which peaked by 74 h, FS showed a rapid increase in N₂ levels, peaking above 9 × 10^3^ nmol g⁻¹ dry soil by 26 h which is quite early in the incubation.

The difference in the accumulation area under the dynamic curve of N_2_ in FS and BS was significant (*p* < 0.001) throughout the incubation in all groups. The total N_2_ accumulation area in FS was 1.11–9.16 times greater than that in BS depending on the group (Fig. S4a-c).

Furthermore, in NSC and FSB groups (Fig. S4d-e), area under the dynamic curve of N_2_O + N_2_ increased over time for both FS and BS. The FS consistently exhibited higher accumulation compared to BS, with significant (*p* < 0.001) differences observed throughout from 50 h. For the BSB group (Fig. S4f), though N_2_O + N_2_ accumulation was higher for FS but the differences were nonsignificant. The total accumulation area of N_2_O + N_2_ for FS was 1.06, 3.6, and 1.04 times greater than that of BS in NSC, FSB, and BSB, respectively.

The N₂O/(N₂O + N₂) ratio was significantly (*p* < 0.001) higher in BS than in FS across all groups (Table 2). These results highlight significant differences in N₂O and N₂ accumulation dynamics between FS and BS across the different treatments. The two-way ANOVA results (Table S3) further confirm the significant effects of both soil types (FS and BS) and treatments (whether inoculation with FS- or BS-derived bacteriomes [FSB, BSB] or leaving the soils untreated [NSC]), with notable interactions influencing N₂O accumulation across the treatments.

**Table 2:** N_2_O ratios calculated as N_2_O/N_2_O + N_2_ based on the area under the curve (AUC) for N₂O relative to the total gas production (N₂O + N₂) over the entire incubation period and displayed as mean values with SEM from triplicate vials per treatment

	FS	BS	Sig
NSC	0.092 ± 0.011	0.631 ± 0.014	***
FSB	0.007 ± 0.001	0.608 ± 0.012	***
BSB	0.025 ± 0.001	0.08 ± 0.004	**

Sig = Significance. ** *p* ≤ 0.003; *** *p* ≤ 0.001

### Comparison of denitrification functional genes

**Fig. 2:**
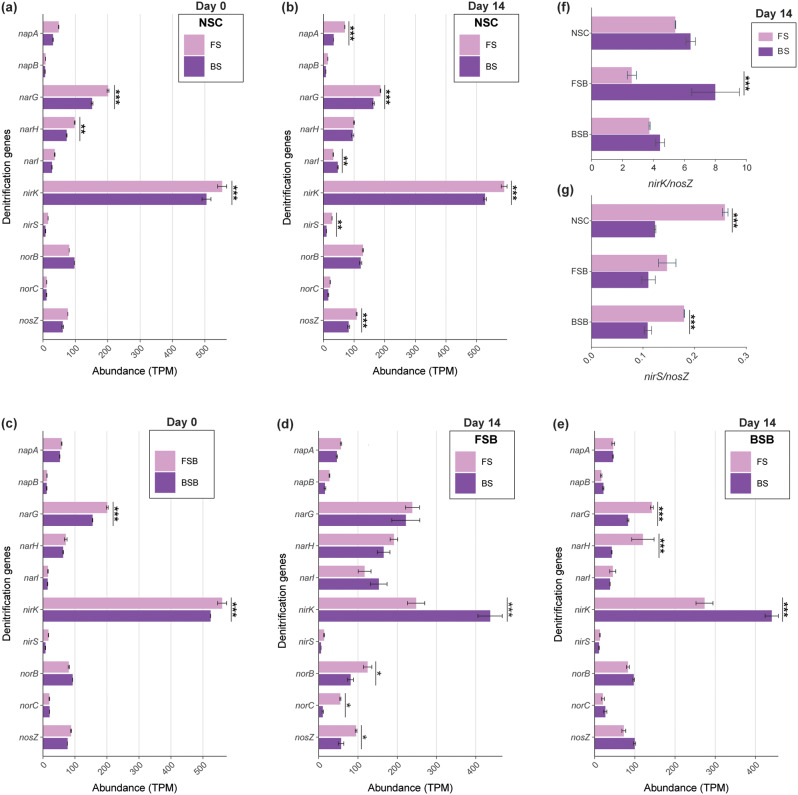
Comparison of shifts in relative abundances of denitrification genes between FS and BS across different treatment groups (NSC, FSB, and BSB) during anoxic incubation from Day 0 to Day 14. **(a)** Gene relative abundances in the NSC group at Day 0. **(b)** Gene relative abundances in the NSC group at Day 14. **(c)** Gene relative abundances of FSB and BSB bacteriome at Day 0. **(d)** Gene relative abundances of FS and BS in the FSB group at Day 14. **(e)** Gene relative abundances of FS and BS in the BSB group at Day 14. **(f)** Differences in the ratios of *nirK*/*nosZ* between FS and BS across the treatment groups. **(g)** Differences in the ratios of *nirS*/*nosZ* between FS and BS across the treatment groups. Gene abundance is expressed in TPM, with data derived from metagenome sequencing. Significant differences between FS and BS are calculated via two-way ANOVA and denoted by asterisks (* *p* < 0.05, ** *p* < 0.01, *** *p* < 0.001)

Based on metagenomic sequence analysis, we evaluated the variations in relative abundances of various denitrification genes between FS and BS in initial soils and samples after 14 days of anoxic incubation. In the NSC group (Fig. 2a-b), the relative abundances of *narG* and *nirK* were significantly higher (*p* < 0.001) in FS compared to BS, both initially and after 14 days of incubation. While the initial relative abundances of *napA*, *nirS*, and *nosZ* were also higher in FS, their differences became statistically significant (*p* < 0.001) after 14 days. Initially, *norB* and *norC* exhibited slightly higher relative abundances in BS; however, this trend reversed after 14 days, though the differences were not statistically significant.

The initial relative abundances of the genes *narG*, *nirK*, *nirS*, and *nosZ* between the FSB and BSB bacteriomes (Fig. 2c) were observed to be higher in the FSB group compared to the BSB group, with the differences in *narG* and *nirK* abundances being statistically significant (*p* < 0.001). After 14 days, within the FSB group (Fig. 2d), the relative abundance of *nirK* was significantly elevated in the BS compared to the FS (*p* < 0.001). Conversely, the relative abundances of *napA*, *napB*, *nirS*, *norB*, *norC*, and *nosZ* were higher in the FS, with the latter three genes showing statistically significant differences (*p* < 0.01). In the BSB group (Fig. 2e), the abundance of *nirK* was also significantly higher in the BS compared to the FS (*p* < 0.001). Although *norB*, *norC*, and *nosZ* exhibited higher abundances in the BS, these differences were not statistically significant. In contrast, the relative abundances of *narG* and *narH* were significantly higher in the FS compared to the BS (*p* < 0.001).

In addition, the ratios of *nirK*/*nosZ* (Fig. 2f) were higher in BS compared to FS across all groups with significant difference (*p* < 0.001) in FSB group. Whereas, the ratios of *nirS*/*nosZ* (Fig. 2g) were higher in FS compared to BS, with significant differences (*p* < 0.01) in the NSC and BSB groups.

The quantified abundances of the four denitrifying functional genes, *narG*, *nirK*, *nirS*, and *nosZ*, determined by qPCR, exhibited trends consistent with the metagenomic sequencing data. After 14 days (Fig. S5a-d), in the NSC group, the copy numbers of all four genes were significantly higher in the FS compared to the BS. In the FSB and BSB groups, the copy numbers of the *nirS* gene were higher in the FS, while the *nirK* gene copies were more abundant in the BS. Notably, the ratios of *nirK* and *nirS* to *nosZ* (Fig. S5e-f) revealed higher *nirK*/*nosZ* ratios in the BS and higher *nirS*/*nosZ* ratios in the FS across all groups, paralleling the trends observed in the metagenomic data.

We observed significant main and interaction effects of soil types and treatments on the abundance of denitrification genes (Table S4), highlighting their influence on denitrification dynamics.

### Bacterial community composition and diversity

**Fig. 3:**
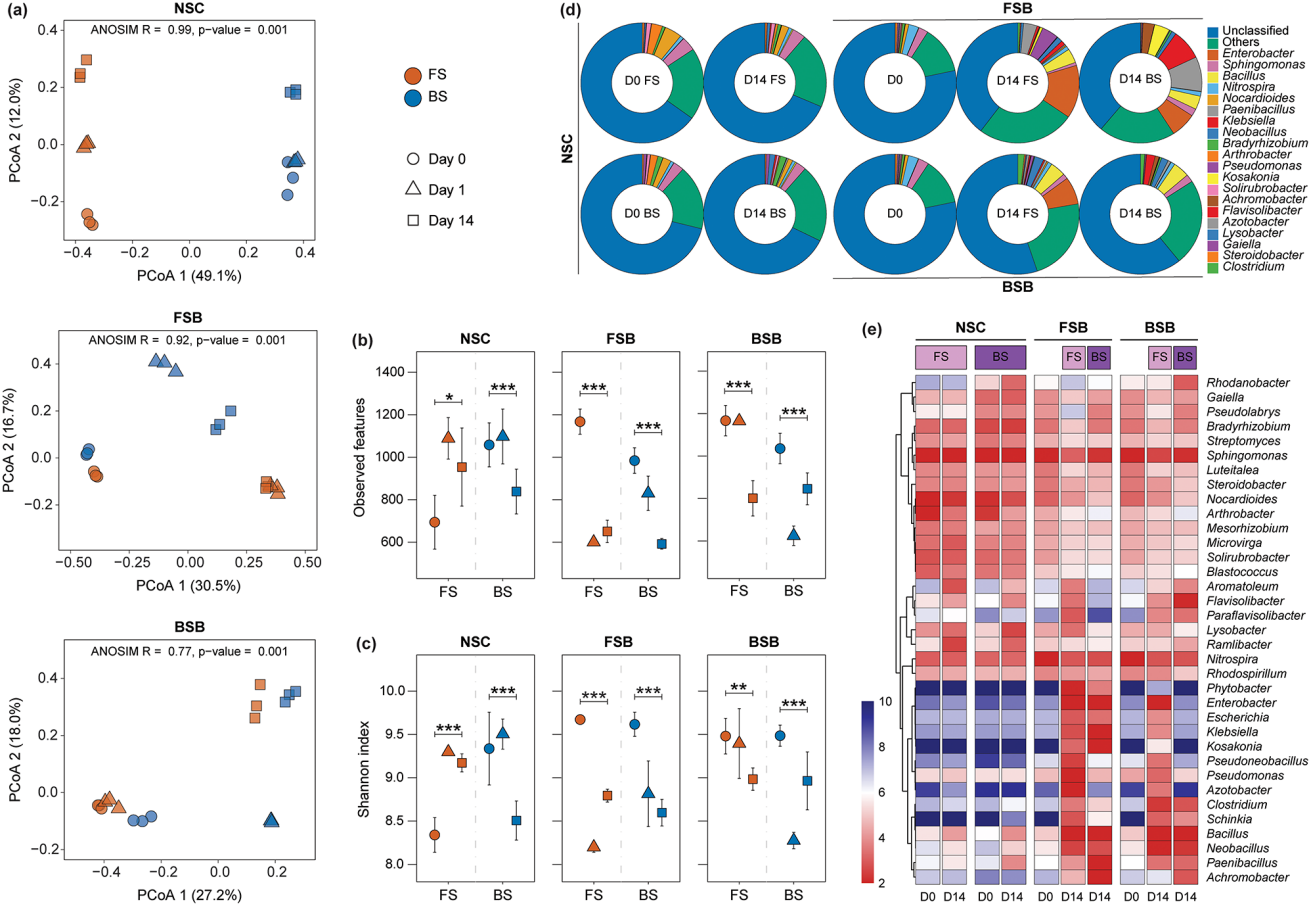
Comparison of microbial community composition and diversity between FS and BS across different treatment groups (NSC, FSB, and BSB) during anoxic incubation over time. **(a)** PCoA plots of microbial communities based on Bray-Curtis dissimilarity in NSC, FSB, and BSB treatment groups. ANOSIM R and *p*-values indicate the strength and significance of differences between FS and BS. **(b)** Observed features and **(c)** Shannon diversity index in NSC, FSB, and BSB groups at different time points, based on 16 S rRNA gene sequencing data. Significant differences between FS and BS are calculated via the Kruskal-Wallis test and denoted by asterisks (* *p* < 0.05, ** *p* < 0.01, *** *p* < 0.001). **(d)** Relative abundance of dominant bacterial taxa at the genus level in NSC, FSB, and BSB groups over time for FS and BS. **(e)** Heatmap showing variations in the relative abundance of dominant bacterial genera in NSC, FSB, and BSB groups over time for FS and BS, based on metagenome sequencing data. Color intensity represents the log-transformed relative abundance of each genus

The PCoA plots (Fig. 3a) based on the Bray-Curtis distance of the 16S rRNA gene V3-V4 region sequences, illustrate the variations in microbial community composition among the groups. The ANOSIM results indicates statistically significant (*p* = 0.001) differences in the microbial community composition between FS and BS across the groups (*R* = 0.99, 0.92, and 0.77, for NSC, FSB, and BSB, respectively). The ANOSIM values reveal significant temporal shifts within microbial communities, particularly in the inoculated groups (FSB and BSB). The same initial inoculum diverged markedly between FS and BS after 14 days of incubation, highlighting a strong impact of recipient soil on microbial community structure.

The alpha diversity metrics, based on 16S rRNA gene V3-V4 region sequences, including the observed features (Fig. 3b) and Shannon index (Fig. 3c), reveal significant differences between FS and BS across the groups (NSC, FSB, BSB) over time. In the FSB and BSB groups, these features showed a significant decrease (*p* < 0.001) towards the end of incubation. Conversely, the NSC group exhibited significant (*p* < 0.001) increase and decrease in diversity for FS and BS, respectively. These results underscore the differential impacts of the recipient soil environment on microbial diversity and richness over time.

The number of clean metagenome sequence reads in the samples varied from 101,115,944 to 126,434,714. The relative abundance of reads with no BLAST hit ranged from 62.4 to 78.8%, depending on the sampling time (higher in samples at the initial time point) and treatment type. The relative abundance of bacterial phyla varied significantly across treatment groups over time (Fig. S6). Initially, Pseudomonadota, Actinomycetota, and Acidobacteriota were predominant in all groups. By the end of the incubation, the bacterial community composition in the NSC group remained relatively stable. In contrast, the FSB and BSB groups exhibited substantial shifts. Both groups demonstrated a marked decline in the relative abundance of Actinomycetota and Acidobacteriota. In the FSB group, there was a significant increase in the abundance of Pseudomonadota and Bacillota in both FS and BS. Meanwhile, the BSB group showed a substantial increase in Bacteroidota in both FS and BS, and a notable increase in Gemmatimonadota in the BS.

Based on metagenome sequencing data, initially, a substantial proportion of bacterial taxa remained unclassified at the genus level across all treatment groups (NSC, FSB, BSB), with percentages ranging from 65.0 to 78.3% (Fig. 3d). By the end of the incubation period, the proportion of unclassified bacterial communities remained relatively stable in the NSC group (67.6 − 68.5%). However, there was a notable reduction in the FSB (38.8 − 39.7%) and BSB (55.1 − 60.8%) groups. Although several genera such as *Sphingomonas*, *Nitrospira*, *Bradyrhizobium*, and *Nocardioides* remained relatively consistent across the groups over time, significant shifts in microbial community structure were evident (Fig. 3e). In the NSC group, genera such as *Flavisolibacter*, *Aromatoleum*, *Lysobacter*, and *Ramlibacter* showed enrichment in both FS and BS over time. The FSB group displayed a significant increase in the relative abundance of *Flavisolibacter*, *Aromatoleum*, *Lysobacter*, *Pseudomonas*, and *Azotobacter* in FS, while *Pseudolabrys* and *Bradyrhizobium* were enriched in BS. In the BSB group, there was a marked increase in the abundance of *Flavisolibacter*, *Aromatoleum*, *Clostridium*, *Bacillus*, *Neobacillus*, and *Paenibacillus* in both FS and BS. Additionally, *Pseudomonas*, *Azotobacter*, *Enterobacter*, *Klebsiella*, and *Kosakonia* were particularly enriched in FS, whereas *Achromobacter* was notably enriched in BS. These observations indicate a shift in taxon composition under different soil conditions.

### Comparison of denitrifying community composition

**Fig. 4:**
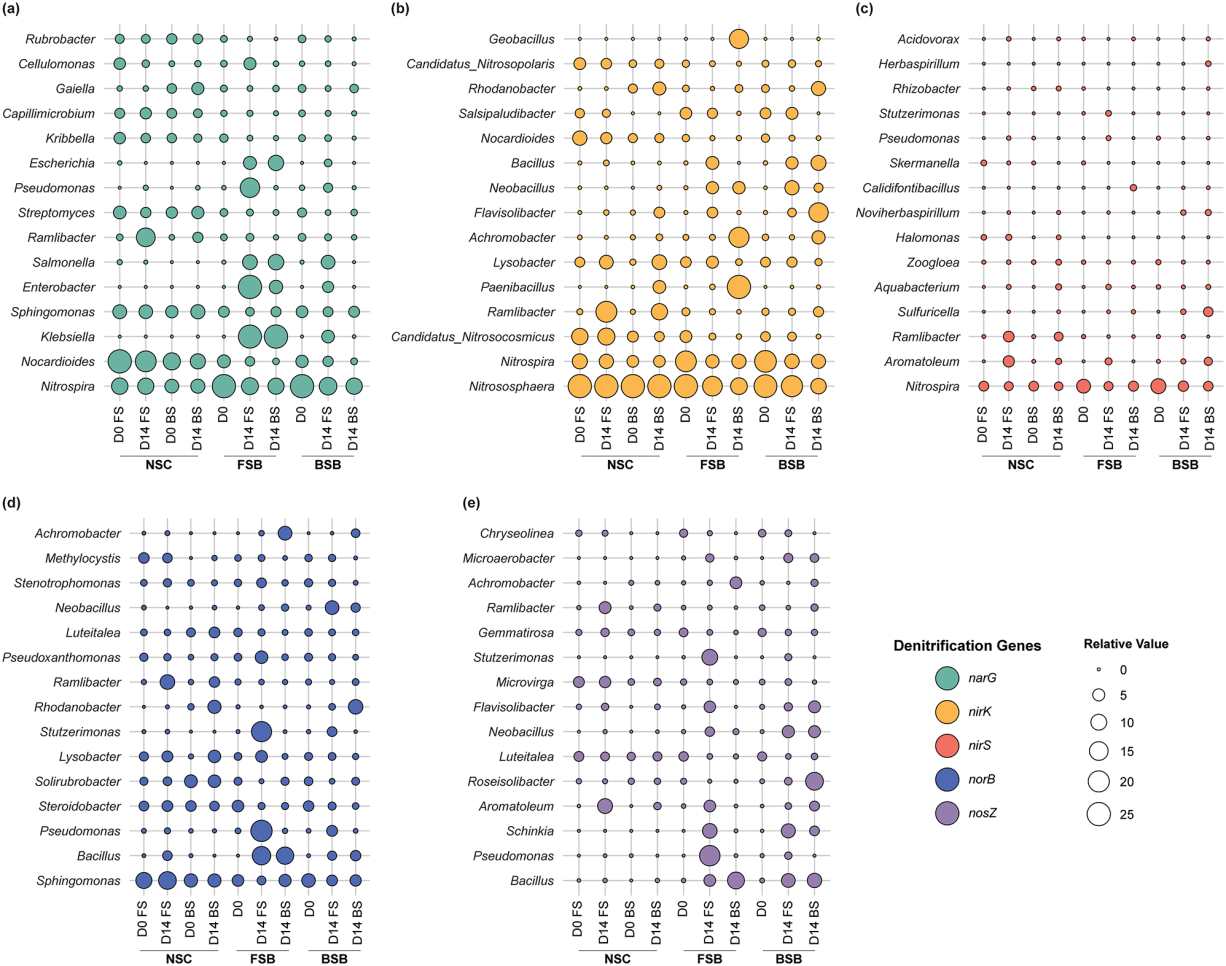
Shifts in the relative abundance of dominant denitrifying bacterial genera between FS and BS across different treatment groups (NSC, FSB, and BSB) over time during anoxic incubation. **(a)** Relative abundance of dominant *narG* gene-containing bacterial genera. **(b)** Relative abundance of dominant *nirK* gene-containing bacterial genera. **(c)** Relative abundance of dominant *nirS* gene-containing bacterial genera. **(d)** Relative abundance of dominant *norB* gene-containing bacterial genera. **(e)** Relative abundance of dominant *nosZ* gene-containing bacterial genera. The size of the circles represents the relative abundance of each denitrifying taxon, while different colors correspond to different denitrification genes, all based on metagenome sequencing data

Based on the analysis of metagenome sequence data, the taxonomic composition of dominant denitrifying genera exhibited significant shifts across different treatment groups over time (Fig. 4). In the NSC group, *Ramlibacter*, which contains the *narG* (Fig. 4a), *nirK* (Fig. 4b), *nirS* (Fig. 4c), *norB* (Fig. 4d), and *nosZ* (Fig. 4e) genes, was enriched in both FS and BS, with a notably higher enrichment in FS. This suggests a greater overall denitrification potential in FS. In the FSB group, potential complete denitrifiers such as *Pseudomonas*, which contains *narG*, *nirS*, *norB*, and *nosZ*, and *Stutzerimonas*, which contains *nirS*, *norB*, and *nosZ*, were significantly enriched only in FS. In contrast, *Paenibacillus*, *Achromobacter*, and *Geobacillus*, which contain only *nirK*, were highly enriched in BS. Similarly, in the BSB group, *Neobacillus*, which contains *nirK*, *norB*, and *nosZ*, was predominantly enriched in FS, while *nirK*-containing *Flavisolibacter* and *nosZ*-containing *Roseisolibacter* were highly enriched in BS. In addition, genus *Rhodanobacter*, containing *nirK* and *norB*, was highly enriched only in the BS of both NSC and BSB groups but declined in the FS of BSB, despite having the same initial inoculum. Overall, these taxonomic variations indicate clear shifts in denitrifying genera based on the recipient soil environment.

**Fig. 5:**
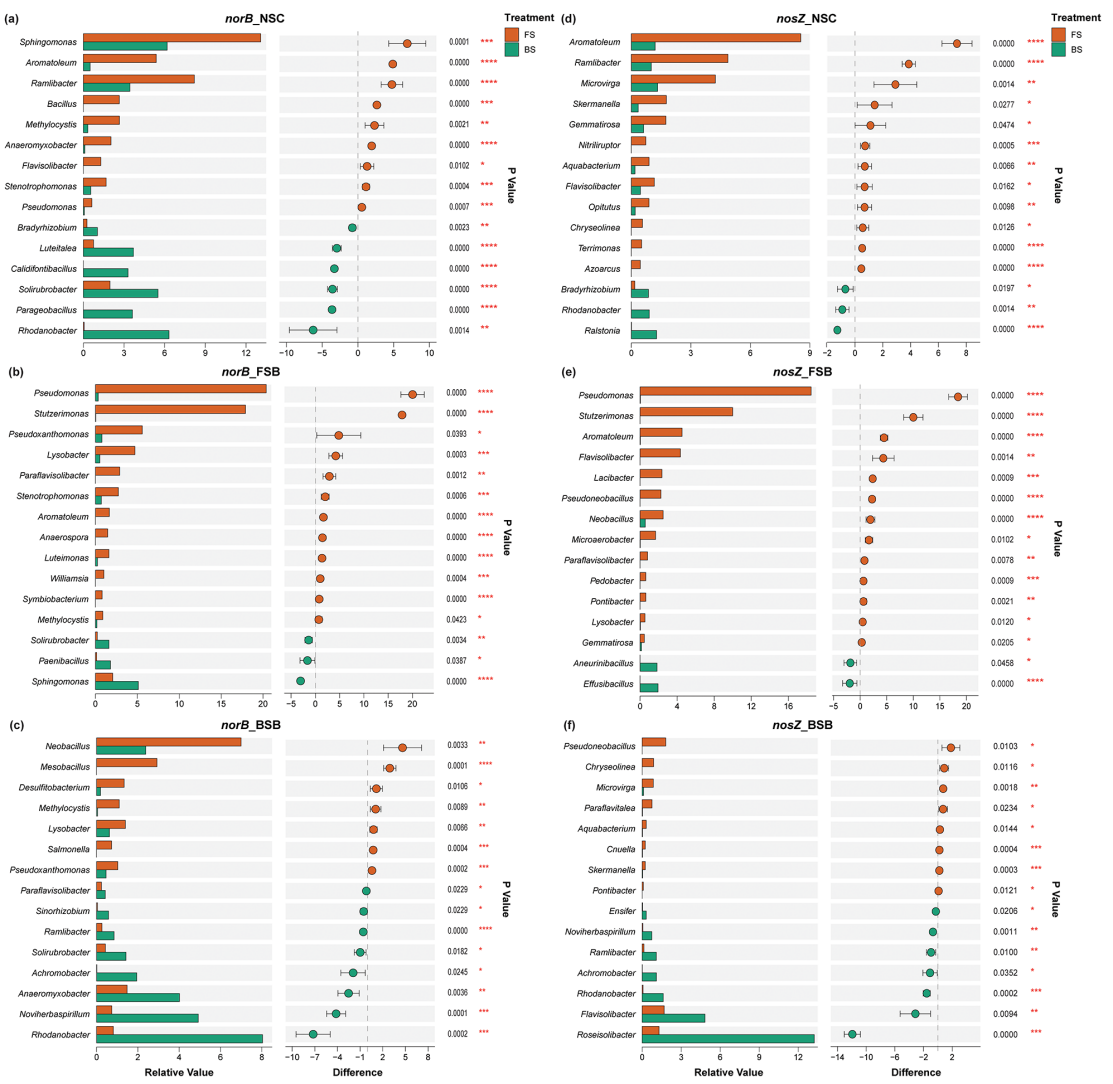
Differences in the relative abundance of denitrifying genera containing *norB* and *nosZ* genes between FS and BS across different treatment groups at the end of anoxic incubation. Relative abundance of bacterial genera in FS and BS containing the *norB* gene in **(a)** NSC, **(b)** FSB, and **(c)** BSB groups. Relative abundance of bacterial genera in FS and BS containing the *nosZ* gene in **(d)** NSC, **(e)** FSB, and **(f)** BSB groups. The left panels display bar plots of the relative abundance of each genus, while the dot plots in the right panels show the confidence intervals (CI) for the differences in relative abundance between FS and BS. Significant differences are calculated via the Kruskal-Wallis test and denoted by asterisks (* *p* < 0.05, ** *p* < 0.01, *** *p* < 0.001, **** *p* < 0.0001). Data to calculate the differences in the relative abundance of denitrifying genera is based on metagenome sequencing

Further statistical analysis revealed significant differences in the relative abundance of denitrifying genera between FS and BS across all treatment groups (Fig. 5 and Fig. S7). For the *nirK*-based genera, the relative abundance of *Nitrososphaera* and *Aromatoleum* was significantly higher in FS (*p* < 0.001), whereas *Rhodanobacter*, *Flavisolibacter*, and *Bradyrhizobium* were more abundant in BS (Fig. S7a-c). In contrast, for *nirS*-containing genera, *Aromatoleum* and *Pseudomonas* were significantly more abundant in FS (*p* < 0.001). However, no *nirS*-containing genus showed a consistent enrichment in BS across the groups (Fig. S7d-f). Furthermore, similar to the *nirS*-based denitrifiers, *Aromatoleum* and *Pseudomonas*, along with some other genera, appeared as *norB* and *nosZ*-based denitrifying genera with a significantly higher relative abundance in FS (*p* < 0.001). Conversely, *Solirubrobacter* was identified as a *norB*-based denitrifier, and *Rhodanobacter* emerged as both *norB*- and *nosZ*-based denitrifier, predominantly in BS across the groups (Fig. 5a-f). In addition, based on PICRUSt2 predictions from the 16S rRNA gene V3-V4 region sequences, the number of taxa predicted to be harbouring a complete denitrification pathway was higher in FS compared to BS across all treatment groups (Table S5).

**Fig. 6:**
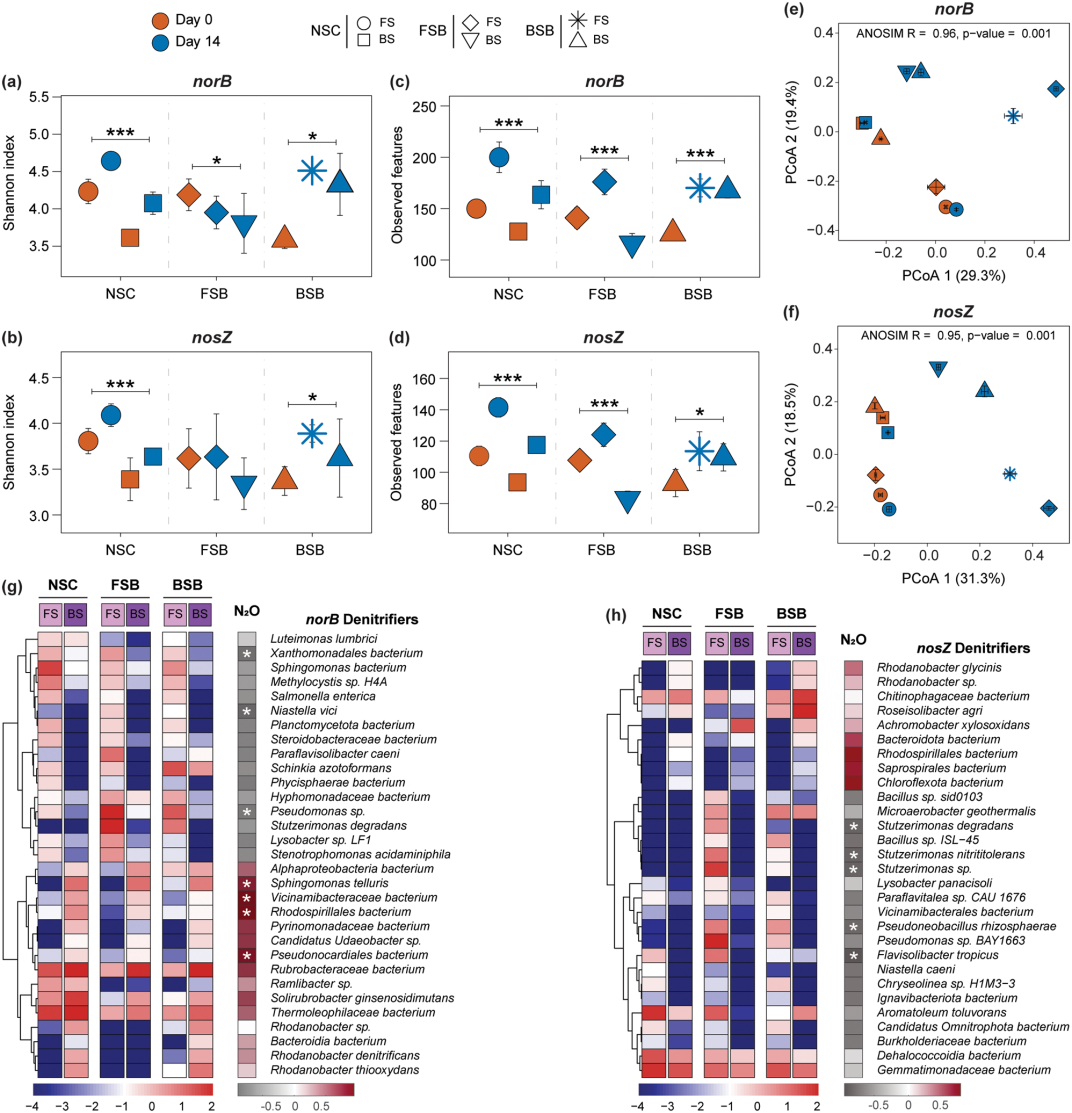
Diversity and composition of key denitrifiers between FS and BS across different treatment groups (NSC, FSB, and BSB) during anoxic incubation over time. Shannon index of the **(a) ***norB* and **(b) ***nosZ* denitrifying community and observed features of the **(c) ***norB* and **(d) ***nosZ* denitrifying community at Day 0 and Day 14 between FS and BS across NSC, FSB, and BSB treatment groups. Significant differences are calculated by the Kruskal-Wallis test and denoted by asterisks (* *p* < 0.05, *** *p* < 0.001). PCoA plots of microbial communities based on the **(e) ***norB* and **(f) ***nosZ* denitrifying community structure at Day 0 and Day 14 across NSC, FSB, and BSB treatment groups. ANOSIM R and p-values indicating significant separation. Heatmaps of LEfSe-derived key **(g) ***norB* denitrifiers and **(h) ***nosZ* denitrifiers, discriminating between FS and BS across NSC, FSB, and BSB groups at Day 14. The colors scheme in the left panel represent the log-transformed relative abundance of the key species in each sample, while those in the right panel denote the R-value of Spearman’s correlation between key species and N_2_O accumulation (* indicates *p* < 0.05). Data used for Fig. 6 is based on metagenome sequencing

The alpha diversity and PCoA of the Bray-Curtis distance, based on metagenomic sequencing results, reveal significant differences in the microbial communities involved in denitrification between FS and BS across the treatment groups. The Shannon index (Fig. 6a and b, and Fig. S8a and b) and observed features (Fig. 6c and d, and Fig. S8c and d) for the *nirK*-, *nirS*-, *norB*-, and *nosZ*-containing bacterial communities significantly (*p* < 0.001) increased towards the end of incubation in both FS and BS of the NSC and BSB groups, with a more pronounced increase in FS. In contrast, the FSB group showed a divergent pattern: the Shannon index for *nirK* (Fig. S8a) and *nirS* (Fig. S8b) based communities increased significantly (*p* < 0.001) in FS but decreased in BS. Furthermore, for the *norB* and *nosZ* communities, the Shannon index decreased in both FS and BS compared to the initial time point. Similarly, the observed features for the FSB group increased significantly (*p* < 0.001) in FS but decreased in BS over time for the *nirS* (Fig. S8d), *norB* (Fig. 6c), and *nosZ* (Fig. 6d) based communities, whereas for the *nirK*-based community (Fig. S8c), a decrease was observed in both FS and BS compared to the initial community. Additionally, the PCoA plots (Fig. 6e and f, and Fig. S8e and f) demonstrated significant differences (*p* = 0.001, ANOSIM test) and clear clustering of denitrifying bacterial communities across the NSC, FSB, and BSB treatment groups between FS and BS over time. These findings elucidate the differential impacts of the recipient soil conditions on the composition and diversity of the denitrifying microbial communities as evolved over time.

### Correlation between the abundance of key denitrifiers and N_2_O accumulation

The output files of the species level abundance data from the metagenome sequencing were used as input for the LEfSe algorithm to identify key *nirK*, *nirS*, *norB*, and *nosZ*-related species in FS and BS across different treatment groups. Subsequently, key species with significant differences in relative abundance between FS and BS were further analyzed to explore their correlation with N_2_O accumulation.

Across the treatment groups, key *nirK*, *nirS*, *norB*, and *nosZ* denitrifying bacterial species (Fig. 6g and h, and Fig. S9a and b) enriched in FS showed either statistically significant (*p* < 0.05) or insignificant but negative correlations with N_2_O emissions. In contrast, those enriched in BS were positively correlated with N_2_O accumulation. Notably, several key denitrifying species related to *Rhodanobacter*, such as *nirK*, *norB*, and *nosZ* based *Rhodanobacter sp.*, *nirK* and *norB* based *Rhodanobacter thiooxydans*, and *nirK* and *nosZ* based *Rhodanobacter glycinis*, were enriched only in the BS of NSC and BSB groups and exhibited a positive correlation with N_2_O emissions (Fig. 6g and h, and Fig. S9a).

On the other hand, key denitrifying species related to *Pseudomonas*, such as *nirS* based *Pseudomonadota bacterium* and *norB* and *nosZ* based *Pseudomonas sp.*, were enriched only in FS across different groups and showed a negative correlation with N_2_O emissions (Fig. 6g and h, and Fig. S9b). Similarly, species related to *Stutzerimonas*, such as key *nirS* based *Stutzerimonas nitrititolerans* (Fig. S9b) and *norB* based *Stutzerimonas degradans* (Fig. 6g), were enriched in the FS of the FSB group and were negatively correlated with N_2_O accumulation. Most importantly, several *nosZ* based key *Stutzerimonas* species, including *Stutzerimonas degradans*, *Stutzerimonas nitrititolerans*, and *Stutzerimonas sp.* (Fig. 6h), showed a significant negative correlation with N_2_O emissions and appeared as potential N_2_O reducing bacteria in the FS of FSB group.

### Impact of recipient soil on denitrifying bacterial community

**Fig. 7:**
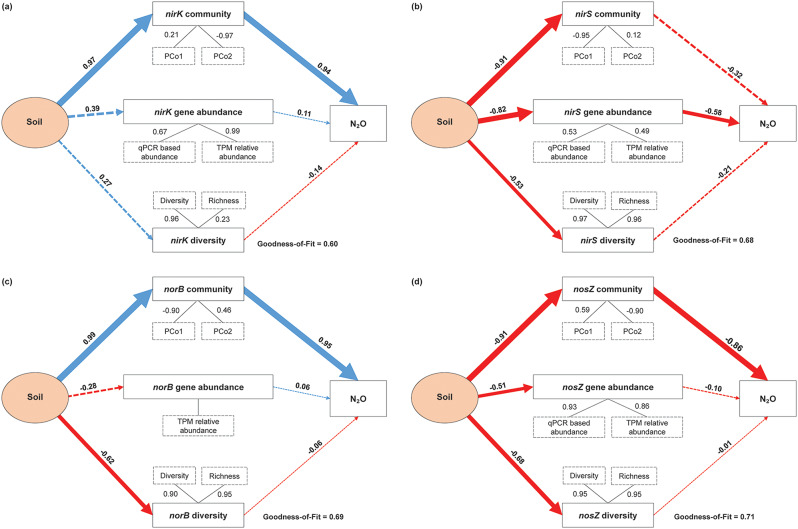
Effects of soil type on denitrifying community diversity and composition. Directed graphs of the PLSPM depicting the impact of soil conditions on the **(a) ***nirK*, **(b) ***nirS*, **(c) ***norB*, and **(d) ***nosZ* denitrifying communities. Each rectangular box represents an observed variable (i.e., measured) or a latent variable (i.e., constructs). Path coefficients are calculated after 1000 bootstraps and are reflected in the solid width of the arrows, with blue and red indicating positive and negative effects, respectively. The loadings for the community structure and diversity that create the latent variables are shown in the dashed rectangles. Dashed arrows indicate that coefficients did not differ significantly (*p* > 0.05). The model is assessed using the GoF statistics

The PLSPM was employed to assess soil influences on denitrifying bacterial communities, specifically *nirK*, *nirS*, *norB*, and *nosZ*. As illustrated in Fig. 7 and Table S6, the interactions between soil environments, gene abundances, diversity indices, and N₂O emissions are evident.

In the *nirK* community model (Fig. 7a), soil positively impacts both the *nirK* community (path coefficient = 0.97) and N₂O emissions (path coefficient = 0.94). Additionally, soil conditions influence *nirK* gene abundance (path coefficient = 0.39) and diversity (path coefficient = 0.27). In contrast, the *nirS* model (Fig. 7b) shows that soil type negatively influences *nirS* community structure (path coefficient = -0.91) and gene abundance (path coefficient = -0.82), with *nirS* gene abundance significantly negatively correlating with N₂O emissions (path coefficient = -0.58).

For the *norB* community (Fig. 7c), soil exhibits a significant positive relation with community structure (path coefficient = 0.99) but negatively impacts diversity (path coefficient = -0.62). The *norB* structure significantly positively correlates with N₂O emissions (path coefficient = 0.95). Lastly, in the *nosZ* model (Fig. 7d), significant negative effects of soil type on community structure (path coefficient = -0.91) and diversity (path coefficient = -0.68) are noted, while the *nosZ* structure negatively impacts N₂O emissions (path coefficient = -0.86). Overall, the goodness-of-fit (GoF) values for the community models range from 0.60 to 0.71, indicating satisfactory fits.

## Discussion

### N_2_O accumulation pattern between two soils

Variations in soil biological and physicochemical properties significantly influence N₂O fluxes in agroecosystems [1, 7, 10]. Similar to Wu et al., [9], our study found substantial differences in N₂O accumulation between FS and BS, which can be attributed to their unique physicochemical characteristics, such as indigenous microbiome and diverse edaphic conditions. These differences are usually reported to lead to varied and often complex responses to N additions in soils [15]. While literature often highlights variations in carbon (C) and N contents as key factors influencing denitrification potential [51], our results suggest otherwise. Despite an equal supply of C and N and maintaining their native microbial communities, the N₂O emission rate of BS was 6.5 times higher than that of FS (Fig. 1a, d). This observation indicates that differences in N₂O accumulation between FS and BS are likely driven by more influential factors, such as their distinct denitrifying bacterial communities [9] and other physicochemical properties. These findings underscore the need for a deeper understanding of the interplay between soil characteristics and microbial communities to more accurately predict and manage N₂O fluxes in agricultural systems.

### Effects of extraction biases on the relevance of extracted bacteriomes to original soil

The cell extraction methods used can extract only a portion of soil cells [31], introducing potential biases in the composition and functioning of the extracted microbial community, such as differences in diversity [52] and N_2_O emission potential [53]. The functional relevance of extracted bacteriomes to their origin soils depends on how accurately they represent soil bacteriomes. Our findings showed high similarity between the composition of bacteriomes from parent soils and extracted cells, despite biases against certain taxa like Acidobacteria and Firmicutes (Fig. S2c, d), consistent with Highton et al. [33]. Despite these constraints, the conserved N_2_O accumulation patterns (Fig. S3) between soils and extracted bacteriomes indicate functional similarity. This underscores the importance of considering both microbial community composition and soil physicochemical properties when studying N_2_O emissions.

### N_2_O Accumulation responses

Several studies have highlighted the influence of soil physicochemical properties and environmental factors as key triggers for microbial-mediated N_2_O emissions from soils [7, 11–14]. In our study, BS consistently exhibited higher N_2_O accumulation, particularly in the NSC (6.50 times), FSB (21.74 times), and BSB (3.26 times) groups, and a significantly higher N_2_O/(N_2_O + N_2_) ratio compared to FS (*p* < 0.001). The observed significant differences in N_2_O and N_2_ accumulation dynamics between FS and BS across various treatment groups highlight the critical role of varying soil properties in these processes.

For the NSC group, the sustained increase in N_2_O production in both BS and FS suggests a stable enhancement in microbial activity or the denitrification process. In the FSB group, the dramatic late increase in N_2_O accumulation in BS indicates an accelerated denitrification process, triggering enhanced N_2_O production. The consistently lower and steady accumulation of N_2_O in FS compared to BS during the incubation period indicates a differential response of the FSB between FS and BS, likely due to variations in microbial community structure [16]. Similarly, the quicker and lower-level accumulation of N_2_O in FS of the BSB group compared to BS also points to divergent underlying denitrification processes contributing to N_2_O emissions.

These findings underscore that N_2_O accumulation is more strongly associated with soil environment rather than bacteriome type. The recipient soil-induced shifts in the denitrification process of the inoculated bacteriome might be influenced mainly by the variation in soil pH along with other properties. As pH can directly or indirectly affect the denitrification process and the denitrifying microbial community, either by inhibiting the assembly and activity of N₂O reductase [12, 16] or by eliciting different responses from various microbial pathways to pH changes [54]. Acidic conditions are known to create an imbalance between N₂O production and consumption, ultimately resulting in net N₂O accumulation [12, 25, 55]. The acidic conditions of BS and the alkaline conditions of FS (Table 1) are likely significant factors contributing to the observed N₂O accumulation patterns.

### Effects on denitrifying functional genes

Our study supports the established correlation between N_2_O emissions in soils and denitrifying genes, notably *nirK* and *nosZ*, which have shown strong positive and negative correlations with potential N_2_O emissions [12, 55, 56]. Soils with high pH and clayey texture exhibit more robust denitrification potential compared to acidic and sandy soils owing to their ability to retain water and organic matter, creating more anaerobic microsites necessary for denitrification which create favourable conditions for denitrifying bacteria [57, 58]. Besides, the anoxic FS condition has been previously indicated as more favourable environment for the accelerated complete denitrification process [59].

Consistent with previous research [9], we observed higher relative abundances of *narG*, *nirK*, *nirS*, and *nosZ* in clayey and alkaline FS, indicating a more robust denitrification potential in these soils compared to sandy and acidic BS in the NSC. This trend was further substantiated in the FSB group, where the initial relative abundances of *narG* and *nirK* were significantly higher in FS. However, towards the end of the incubation, we observed a significant elevation in *nirK* abundance in BS, and higher abundances of *nirS*, *norB*, *norC*, and *nosZ* in FS, which suggest differential microbial adaptations and gene regulations in response to the conditions of the recipient soil. Notably, in the BSB group, the consistently higher *nirK* abundance in BS points to microbial adaptation favouring this gene under anoxic conditions.

The qPCR data (Fig. S5) corroborate our metagenomic findings (Fig. 2), reflecting similar trends in gene abundance. The *nirS* gene, known to be sensitive to soil variation such as pH [60], showed lower abundance in the acidic BS conditions, particularly in the high-pH-adapted bacteriome of the FSB group. Conversely, the abundance of *nirK*, which is positively correlated with N_2_O emissions across various soils [55, 61], was higher in BS, aligning with its higher N_2_O emissions. Furthermore, higher *nirK*/*nosZ* ratios in BS suggest a shift towards incomplete denitrification processes, while higher *nirS*/*nosZ* ratios in FS indicate a balance favouring a complete denitrification pathway. A study by Liu et al. [62] identified a negative correlation between N_2_O flux and the copy numbers of *nirS* and *nosZ* genes. They also found that an increase in the numbers of these genes accelerated the reduction of N_2_O to N_2_.

These findings highlight significant variations in denitrification gene abundances for the same bacteriome between FS and BS conditions, underscoring the influence of soil properties on microbial community structure and denitrification potential.

### Divergent bacterial communities caused by recipient soils

Microbial diversity and community composition are crucial for system stability and microbial functioning [26, 27, 63]. Specialized functions such as denitrification, nitrification, and pesticide mineralization are performed by specific microbial groups and are sensitive to changes in microbial composition and diversity [63, 64].

In this study, we observed variations in microbial community composition and diversity between FS and BS across different treatment groups, highlighting the complex dynamics of microbial adaptation to the exposed environmental conditions [65]. Previous studies have shown that inoculated microbial communities in sterile soils often lose taxonomic diversity compared to their origin soils [66, 67]. Consistent with these findings, the drastic decrease over time in observed features and Shannon index values in both FSB and BSB groups, compared to the NSC group, underscores the profound sensitivity of the inoculated microbiomes to sterile anoxic soil conditions. In contrast, the significant increase in diversity indices in FS and the decrease in BS over time within the NSC group indicate the stability and robustness of FS microbial communities in an anoxic soil environment compared to BS.

Shifts in taxonomic composition over time reveal distinct patterns of microbial succession. The initial dominance and subsequent decline of Actinomycetota and Acidobacteriota in the FSB and BSB groups reflect changing microbial dynamics. Microbial succession in response to changes in the soil environment is a dynamic process where microbial community composition and functioning shift to promote a more resilient soil ecosystem [68]. However, the microbial inoculums can also significantly influence community composition [69]. This is evident from the notable increase in Pseudomonadota and Bacillota in the FSB group, and the rise in Bacteroidota and Gemmatimonadota in the BSB group, highlighting the differential enrichment of specific phyla driven by distinct microbial inoculums.

The enrichment of genera such as *Pseudomonas*,* Clostridium*, and *Bacillus* across groups suggests that these genera play key roles in microbial adaptation to anoxic conditions and are integral to nutrient cycling and energy flow in anoxic environments [70–72]. Additionally, the enrichment of specific genera like *Azotobacter*, *Enterobacter*, *Klebsiella*, and *Kosakonia* in FS and *Achromobacter* in BS demonstrates specific soil dependent responses of microbial communities. In short, the significant dynamic shifts in microbial populations and the enrichment of specific taxa highlight the complex interplay between microbial communities and their environments.

### Linkage between shifts in Denitrifying guilds and N_2_O Emission

In soils, denitrifying guilds, functional groups of bacteria harboring specific denitrification genes, play a crucial role in regulating the balance between N₂O production and consumption, ultimately influencing N₂O fluxes [19, 63, 73]. Our study observed significant shifts in the taxonomic composition of denitrifying bacterial communities between FS and BS across various treatment groups, underscoring the strong influence of soil properties on denitrification potential and N₂O emissions.

Previous research has shown that soil properties anchor distinct *nirK*- and *nirS*-based denitrifier communities [74–76]. Our study found few *nirS*-based denitrifiers, mainly in FS, suggesting these denitrifiers are more sensitive to changes such as soil type, moisture, and pH [60, 76]. In the NSC group, *Ramlibacter*, a genus with a comprehensive set of denitrification genes (*narG*, *nirK*, *nirS*, *norB*, and *nosZ*), was more enriched in FS, indicating that FS conditions favor higher denitrification potential. This trend was consistent across groups, with FS generally supporting a more diverse and active denitrifying community than BS. Notably, in the FSB group, complete denitrifiers with *nirS*, *norB*, and *nosZ* genes, such as *Pseudomonas* and *Stutzerimonas*, were significantly enriched in FS. These genera negatively correlated with N₂O emissions, indicating their role in reducing N₂O accumulation through complete denitrification pathways. Previous studies have reported the N₂O reducing potential of *Pseudomonas* and *Stutzerimonas* [71, 77–79], highlighting their significance as complete denitrifiers.

For example, inoculating two *Stutzerimonas stutzeri* strains, NRCB010 and NRCB025, promoted tomato growth and reduced N₂O emissions in agricultural soils, with NRCB010 decreasing emissions by 38.7–52.2% and NRCB025 by 76.6% [77]. Another study noted the high potential of the NRCB010 strain (previously known as *Pseudomonas stutzeri*) for improving crop productivity and mitigating N₂O emissions in agricultural soils [79]. Furthermore, a recent study by Zhang et al. [80] identified denitrifying microorganisms in the deep vadose zone of an intensive agricultural area in China, revealing high denitrification activity in two complete denitrifiers belonging to the genus *Pseudomonas*. These findings suggest that bacteria of these genera may play a central role in the denitrification process in FS. Further, enrichment of potential *nosZ*-containing bacteria could help eliminate N₂O emissions [19, 81]. Our study identified significant enrichment of *nosZ*-containing genera, including *Aromatoleum*, *Ramlibacter*, *Pseudomonas*, and *Stutzerimonas*, in FS across groups. The delay in the onset of denitrification in BS of the FSB group, with extensive N₂O accumulation, might be attributed to the loss of such potential denitrifiers.

Conversely, the relative abundance of *nirK* genes in the FSB and BSB groups was higher in BS than in FS, potentially facilitating increased N₂O generation in BS. BS also showed higher enrichment of *nirK*-containing taxa such as *Paenibacillus* and *Geobacillus*. Additionally, *Rhodanobacter*, containing both *nirK* and *norB* genes, emerged as the dominant denitrifier in BS, with a strong positive correlation between these biomarker species, particularly *Rhodanobacter* and *Achromobacter*, and N₂O accumulation. Previous studies identified *Rhodanobacter* as a prevalent denitrifier in agricultural BS [9, 82]. Specifically, 18 *Rhodanobacter* isolates from Wu et al. [9] and three from Lycus et al. [8] demonstrated significant roles in N₂O accumulation due to their truncated denitrification genes and lack of N₂O reduction capacity. *Achromobacter*, also containing the *nirK* gene [83], has been shown to enhance N₂O spillover [84]. Thus, the enrichment of *nirK*- and *norB*-containing *Rhodanobacter* and *Achromobacter* as dominant denitrifiers may impede the complete denitrification process, potentially leading to increased N₂O emissions in BS across the treatment groups. However, in the FS of the BSB group, where the relative abundance of *Rhodanobacter* dropped significantly, this effect might be mitigated.

Alpha and beta diversity analyses further underscored the structural and compositional differences in denitrifying communities between FS and BS. PCoA plots indicate a significant temporal divergence in the denitrifying communities between the FS and BS across groups. For the inoculant groups, the denitrifying communities of the same inoculated bacteriome were significantly divergent between FS and BS over time and were structurally more related to recipient soil microbiome instead of the inoculum. Our observations align with findings from a previous study where the soil communities possessed functional and taxonomical structures more related to the recipient sterile soils after inoculation and incubation [85]. Further, FS showed significant increases in Shannon index and observed features across most groups, suggesting that FS promotes a more diverse and complex microbial community. This increased diversity may enhance the overall functional resilience and efficiency of the denitrification process, potentially reducing N₂O emissions. In contrast, the FSB group’s pattern of increased diversity in FS but decreased diversity in BS highlights the varying impacts of recipient soil on denitrifying microbial community dynamics. As microbial diversity is crucial for system stability and microbial functioning [27], the decline in diversity in BS could lead to a less stable and low efficiency in denitrification process, contributing to higher N₂O emissions. In short, similar to literature where the composition of *nirK*, *nirS*, and *nosZ*-based denitrifiers influences the enzymatic activity of the denitrification process [74, 75, 86]. Distinct *nirK* and *norB*, and *nirS* and *nosZ* based denitrifiers appeared as important groups for the differential N₂O accumulation patterns in our study.

PLSPM analysis provided a holistic view of the interactions between soil propeties, denitrifying bacterial communities, gene abundances, and N₂O emissions. The significant positive effect of soil condition on *nirK* and *norB* communities and their correlation with increased N₂O emissions highlights the role of these genes in N₂O production. Conversely, the negative influence of soil condition on *nirS* and *nosZ* communities, along with their negative correlations with N₂O emissions, suggests that enhancing the abundance and activity of these communities could be a strategy for reducing N₂O emissions.

In summary, our study highlights the intricate relationships between soil properties, denitrifying bacterial community composition, and N₂O emissions. Our results suggest that the recipient soil properties alter the denitrifier composition of the inoculated bacteriomes, ultimately affecting the activities of denitrifying enzymes and resulting in different patterns of N₂O emission.

## Conclusions

This study reveals the crucial role of soil properties as distal drivers in shaping proximal denitrifying bacterial communities and their influence on N₂O emissions. The variation in N₂O accumulation between FS and BS, even when inoculated with the same microbiome, highlights the impact of recipient soil characteristics on microbial dynamics. BS favoured *nirK*-based denitrifiers like *Rhodanobacter*, leading to incomplete denitrification and higher N₂O emissions, whereas FS supported a more diverse range of denitrifiers, such as *Pseudomonas* and *Stutzerimonas*, promoting complete denitrification and lower emissions. These findings underscore the importance of soil properties in modulating microbial structure and function, which is vital for strategies to optimize denitrification and reduce N₂O emissions in agricultural systems. Future research should explore manipulating soil microbial communities with targeted denitrifiers and applying multi-omics approaches to better understand microbial functions. Expanding the range of soil samples from diverse locations would also strengthen the generalizability of these conclusions, offering a basis for targeted interventions that enhance sustainable agricultural practices and contribute to greenhouse gas mitigation.

## Electronic supplementary material

Below is the link to the electronic supplementary material.

Supplementary Material 1

## Data Availability

The raw Illumina sequence data of the 16S rRNA gene V3-V4 region and metagenomic sequences is available in the GenBank Sequence Read Archive (SRA) database of the National Center for Biotechnology Information (NCBI) under BioProject accession number PRJNA992239 and PRJNA1138288, respectively.
