# Supplementary material for: Soil properties drive nitrous oxide accumulation patterns by shaping denitrifying bacteriomes
Source: Environ Microbiome. 2024 Nov 21;19:94. doi: 10.1186/s40793-024-00643-9 (PMC11580698; doi:10.1186/s40793-024-00643-9)
Supplement: Supplementary file 1 — Supplementary Material 1 [file 40793_2024_643_MOESM1_ESM.docx]

**Soil properties drive nitrous oxide accumulation patterns by shaping denitrifying bacteriomes**

**Saira Bano, Qiaoyu Wu, Siyu Yu, Xinhui Wang, Xiaojun Zhang***

State Key Laboratory of Microbial metabolism, Joint International Research Laboratory of Metabolic & Developmental Sciences, and School of Life Sciences & Biotechnology, Shanghai Jiao Tong University, Shanghai, 200240, China

*Corresponding author: Dr. Xiaojun Zhang

State Key Laboratory of Microbial Metabolism, School of Life Sciences & Biotechnology, Shanghai Jiao Tong University, Shanghai, 20040, PR China.

Tel: +86 21-34204878; Fax: +86 21-34204878;

E-mail: [xjzhang68@sjtu.edu.cn](mailto:xjzhang68@sjtu.edu.cn)

**Supplementary Information**

1. Supplementary materials and methods
2. Supplementary tables
3. Supplementary figures
4. **Supplementary Materials and Methods**
   1. **Soil physicochemical properties analysis**

The nitrate-nitrogen content of the soil was determined by using 1 molar KCl solution to extract the soil nitrate and then automatic chemical discontinuity analyser was used to analyse it. Further, ammonium-nitrogen was assessed by the indophenol blue method and soil nitrite nitrogen was estimated by N- (1-naphthyl) -ethylenediamine dihydrochloride spectrophotometric method [1]. pH of the soils was measured by making 1:5 suspension of soil and detected by pH meter (Mettler-Toledo, Switzerland). The moisture content of the soils was determined by the oven drying method at 105 °C.

Water holding capacity (WHC) was determined using the funnel method, following the procedure outlined previously **[2],** with some modifications. Briefly a specific amount of fresh soil was weighed and placed into a funnel lined with filter paper. The bottom of the funnel was stuffed with cotton, and the funnel was immersed in water, ensuring the water surface was 3-4 cm above the bottom of the funnel. The soil was left to absorb water for 2 hours. Afterward, the funnel was removed, and the cotton was taken out, allowing the water to drain into a bottle for 6 hours.

The moistened soil was then transferred to a tin foil-lined container and weighed. It was dried at 105°C for 12 hours, and the dry weight was measured. The saturated water-holding capacity was calculated using the formula:

$$WHC \%=(c\mathbf{-}a)(b\mathbf{-}c)\boldsymbol{\times}100$$

Where, a = weight of tin foil, b = weight of tin foil + moist soil, and c = weight of tin foil + dried soil

- 1. **Primers used for quantitative PCR**

10 ng/µl DNA was used as a template to quantify soil denitrification functional genes *narG*, *nirS*, *nirK* and *nosZ*. Each 96-well plate contained a plasmid standard DNA sample and a negative control, with 3 technical replicates for each sample. At the end of the cycle, melting curve analysis was performed to detect the specificity of PCR amplification. The primers used for the amplification of the functional genes are described in Table S2**.**

1. **Supplementary tables**

**Table S1** Information about the soils used in the current study adopted from Wu et al. [3].

| **Information** | **FS** | **BS** |
| --- | --- | --- |
| Latitude and longitude | 40^o^18′ N,116^o^10′ E | 43^o^18′ N,124^o^14′ E |
| Major cropping | Wheat, Maize | Maize |
| Manure addition | Winter wheat 300 kg N/ha, Summer corn 260 kg N/ha | N: 234 kg N/ha, P_2_O_5_: 108 kg/ha, K_2_O:108 kg/ha |
| Nitrogen fertilizers inputs | Conventional fertilization | Compound fertilizer |

**Table S2** Primers used for quantitative PCR

| **Gene** | **Primer** | **Primer sequence** | **Fragment size (bp)** | **References** |
| --- | --- | --- | --- | --- |
| *nirK* | nirK1040 | GCCTCGATCAGRTTRTGGTT | 473 | [4] |
|  | FlaCu | ATCATGGTSCTGCCGCG |  |  |
| *nirS* | cd3aF | GTS AAC GTSAAG GAR ACS GG | 425 | [5] |
|  | R3cd | GAS TTC GGRTGSGTC TTG A |  |  |
| *narG* | narG-f | TCGCCSATYCCGGCSATGTC | 173 | [6] |
|  | narG-r | GAGTTGTACCAGTCRGCSGAYTCSG |  |  |
| *nosZ* | nosZ–2f | CGCRACGGCAASAAGGTSMSSGT | 267 | [7] |
|  | nsoZ-2r | CAKRTGCAKSGCRTGGCAGAA |  |  |

**Table S3** List of taxa predicted based on PICRUSt2 prediction to have a complete and partial denitrification pathway in each treatment based on 16S rRNA gene V3-V4 region sequences. Denitrification genes highlighted green were predicted to be associated with corresponding taxon. The taxa highlighted blue were predicted to harbour all the genes necessary for the full denitrification. The rest of the taxa were predicted as partial denitrifiers where, taxa highlighted gray and orange were predicted as potential N_2_O producers and reducers, respectively.

| Treat. | Soil | Predicted denitrifying taxa | Predicted denitrification genes | | | | | | | Taxon RA % |
| --- | --- | --- | --- | --- | --- | --- | --- | --- | --- | --- |
|  |  |  | NO₃→NO₂ | | | NO₂→NO | NO→N₂O | | N₂O→N₂ |  |
| NSC | FS | Uncultured | *narG* | *narH* | *narl* | *nirK* | *norB* | *norC* | *nosZ* | 20.97 |
|  |  |  |  | *napA* | *napB* | *nirS* |  |  |  |  |
|  |  | *g__Lysobacter* | *narG* | *narH* | *narl* | *nirK* | *norB* | *norC* | *nosZ* | 4.67 |
|  |  |  |  | *napA* | *napB* | *nirS* |  |  |  |  |
|  |  | *g__Ramlibacter* | *narG* | *narH* | *narl* | *nirK* | *norB* | *norC* | *nosZ* | 4.48 |
|  |  |  |  | *napA* | *napB* | *nirS* |  |  |  |  |
|  |  | *g__Anaeromyxobacter* | *narG* | *narH* | *narl* | *nirK* | *norB* | *norC* | *nosZ* | 2.38 |
|  |  |  |  | *napA* | *napB* | *nirS* |  |  |  |  |
|  |  | *f__Nitrosomonadaceae* | *narG* | *narH* | *narl* | *nirK* | *norB* | *norC* | *nosZ* | 2.27 |
|  |  |  |  | *napA* | *napB* | *nirS* |  |  |  |  |
|  |  | *g__Noviherbaspirillum* | *narG* | *narH* | *narl* | *nirK* | *norB* | *norC* | *nosZ* | 2.14 |
|  |  |  |  | *napA* | *napB* | *nirS* |  |  |  |  |
|  |  | *g__Azoarcus* | *narG* | *narH* | *narl* | *nirK* | *norB* | *norC* | *nosZ* | 2.03 |
|  |  |  |  | *napA* | *napB* | *nirS* |  |  |  |  |
|  |  | *g__Bacillus* | *narG* | *narH* | *narl* | *nirK* | *norB* | *norC* | *nosZ* | 1.84 |
|  |  |  |  | *napA* | *napB* | *nirS* |  |  |  |  |
|  |  | *g__Gemmatimonas* | *narG* | *narH* | *narl* | *nirK* | *norB* | *norC* | *nosZ* | 1.31 |
|  |  |  |  | *napA* | *napB* | *nirS* |  |  |  |  |
|  |  | *g__Nitrospira* | *narG* | *narH* | *narl* | *nirK* | *norB* | *norC* | *nosZ* | 1.23 |
|  |  |  |  | *napA* | *napB* | *nirS* |  |  |  |  |
|  |  | *f__Comamonadaceae* | *narG* | *narH* | *narl* | *nirK* | *norB* | *norC* | *nosZ* | 1.15 |
|  |  |  |  | *napA* | *napB* | *nirS* |  |  |  |  |
|  | Total |  |  |  |  |  |  |  |  | 44.47 |
|  | BS | Uncultured | *narG* | *narH* | *narl* | *nirK* | *norB* | *norC* | *nosZ* | 22.36 |
|  |  |  |  | *napA* | *napB* | *nirS* |  |  |  |  |
|  |  | *g__Rhodanobacter* | *narG* | *narH* | *narl* | *nirK* | *norB* | *norC* | *nosZ* | 7.64 |
|  |  |  |  | *napA* | *napB* | *nirS* |  |  |  |  |
|  |  | *g__Gemmatimonas* | *narG* | *narH* | *narl* | *nirK* | *norB* | *norC* | *nosZ* | 4.05 |
|  |  |  |  | *napA* | *napB* | *nirS* |  |  |  |  |
|  |  | *f__Comamonadaceae* | *narG* | *narH* | *narl* | *nirK* | *norB* | *norC* | *nosZ* | 2.92 |
|  |  |  |  | *napA* | *napB* | *nirS* |  |  |  |  |
|  |  | *f__Nitrosomonadaceae* | *narG* | *narH* | *narl* | *nirK* | *norB* | *norC* | *nosZ* | 2.34 |
|  |  |  |  | *napA* | *napB* | *nirS* |  |  |  |  |
|  |  | *g__Luteimonas* | *narG* | *narH* | *narl* | *nirK* | *norB* | *norC* | *nosZ* | 2.32 |
|  |  |  |  | *napA* | *napB* | *nirS* |  |  |  |  |
|  |  | *g__Rubrobacter* | *narG* | *narH* | *narl* | *nirK* | *norB* | *norC* | *nosZ* | 1.42 |
|  |  |  |  | *napA* | *napB* | *nirS* |  |  |  |  |
|  |  | *g__Flavisolibacter* | *narG* | *narH* | *narl* | *nirK* | *norB* | *norC* | *nosZ* | 1.16 |
|  |  |  |  | *napA* | *napB* | *nirS* |  |  |  |  |
|  | Total |  |  |  |  |  |  |  |  | 44.21 |
| FSB | FS | *g__Enterobacter* | *narG* | *narH* | *narl* | *nirK* | *norB* | *norC* | *nosZ* | 26.87 |
|  |  |  |  | *napA* | *napB* | *nirS* |  |  |  |  |
|  |  | *f__Enterobacteriaceae* | *narG* | *narH* | *narl* | *nirK* | *norB* | *norC* | *nosZ* | 17.14 |
|  |  |  |  | *napA* | *napB* | *nirS* |  |  |  |  |
|  |  | *g__Pseudomonas* | *narG* | *narH* | *narl* | *nirK* | *norB* | *norC* | *nosZ* | 14.50 |
|  |  |  |  | *napA* | *napB* | *nirS* |  |  |  |  |
|  |  | Uncultured | *narG* | *narH* | *narl* | *nirK* | *norB* | *norC* | *nosZ* | 12.41 |
|  |  |  |  | *napA* | *napB* | *nirS* |  |  |  |  |
|  |  | *g__Bacillus* | *narG* | *narH* | *narl* | *nirK* | *norB* | *norC* | *nosZ* | 10.57 |
|  |  |  |  | *napA* | *napB* | *nirS* |  |  |  |  |
|  |  | *g__Pseudoxanthomonas* | *narG* | *narH* | *narl* | *nirK* | *norB* | *norC* | *nosZ* | 1.27 |
|  |  |  |  | *napA* | *napB* | *nirS* |  |  |  |  |
|  |  | *g__Azoarcus* | *narG* | *narH* | *narl* | *nirK* | *norB* | *norC* | *nosZ* | 1.08 |
|  |  |  |  | *napA* | *napB* | *nirS* |  |  |  |  |
|  |  | *g__Klebsiella* | *narG* | *narH* | *narl* | *nirK* | *norB* | *norC* | *nosZ* | 1.07 |
|  |  |  |  | *napA* | *napB* | *nirS* |  |  |  |  |
|  |  | *g__Kosakonia* | *narG* | *narH* | *narl* | *nirK* | *norB* | *norC* | *nosZ* | 1.05 |
|  |  |  |  | *napA* | *napB* | *nirS* |  |  |  |  |
|  | Total |  |  |  |  |  |  |  |  | 85.97 |
|  | BS | *g__Paenibacillus* | *narG* | *narH* | *narl* | *nirK* | *norB* | *norC* | *nosZ* | 18.92 |
|  |  |  |  | *napA* | *napB* | *nirS* |  |  |  |  |
|  |  | *g__Kosakonia* | *narG* | *narH* | *narl* | *nirK* | *norB* | *norC* | *nosZ* | 18.61 |
|  |  |  |  | *napA* | *napB* | *nirS* |  |  |  |  |
|  |  | *g__Bacillus* | *narG* | *narH* | *narl* | *nirK* | *norB* | *norC* | *nosZ* | 12.13 |
|  |  |  |  | *napA* | *napB* | *nirS* |  |  |  |  |
|  |  | *g__Enterobacter* | *narG* | *narH* | *narl* | *nirK* | *norB* | *norC* | *nosZ* | 10.04 |
|  |  |  |  | *napA* | *napB* | *nirS* |  |  |  |  |
|  |  | *f__Enterobacteriaceae* | *narG* | *narH* | *narl* | *nirK* | *norB* | *norC* | *nosZ* | 10.01 |
|  |  |  |  | *napA* | *napB* | *nirS* |  |  |  |  |
|  |  | *g__Klebsiella* | *narG* | *narH* | *narl* | *nirK* | *norB* | *norC* | *nosZ* | 5.49 |
|  |  |  |  | *napA* | *napB* | *nirS* |  |  |  |  |
|  |  | *g__Achromobacter* | *narG* | *narH* | *narl* | *nirK* | *norB* | *norC* | *nosZ* | 3.56 |
|  |  |  |  | *napA* | *napB* | *nirS* |  |  |  |  |
|  |  | *g__Pseudomonas* | *narG* | *narH* | *narl* | *nirK* | *norB* | *norC* | *nosZ* | 2.88 |
|  |  |  |  | *napA* | *napB* | *nirS* |  |  |  |  |
|  | Total |  |  |  |  |  |  |  |  | 81.64 |
| BSB | FS | *g__Enterobacter* | *narG* | *narH* | *narl* | *nirK* | *norB* | *norC* | *nosZ* | 20.46 |
|  |  |  |  | *napA* | *napB* | *nirS* |  |  |  |  |
|  |  | *g__Bacillus* | *narG* | *narH* | *narl* | *nirK* | *norB* | *norC* | *nosZ* | 9.06 |
|  |  |  |  | *napA* | *napB* | *nirS* |  |  |  |  |
|  |  | Uncultured | *narG* | *narH* | *narl* | *nirK* | *norB* | *norC* | *nosZ* | 6.43 |
|  |  |  |  | *napA* | *napB* | *nirS* |  |  |  |  |
|  |  | *g__Desulfitobacterium* | *narG* | *narH* | *narl* | *nirK* | *norB* | *norC* | *nosZ* | 2.78 |
|  |  |  |  | *napA* | *napB* | *nirS* |  |  |  |  |
|  |  | *g__Pseudomonas* | *narG* | *narH* | *narl* | *nirK* | *norB* | *norC* | *nosZ* | 1.97 |
|  |  |  |  | *napA* | *napB* | *nirS* |  |  |  |  |
|  |  | *g__Azoarcus* | *narG* | *narH* | *narl* | *nirK* | *norB* | *norC* | *nosZ* | 1.09 |
|  |  |  |  | *napA* | *napB* | *nirS* |  |  |  |  |
|  |  | *g__Gemmatimonas* | *narG* | *narH* | *narl* | *nirK* | *norB* | *norC* | *nosZ* | 1.03 |
|  |  |  |  | *napA* | *napB* | *nirS* |  |  |  |  |
|  | Total |  |  |  |  |  |  |  |  | 42.83 |
|  | BS | *g__Bacillus* | *narG* | *narH* | *narl* | *nirK* | *norB* | *norC* | *nosZ* | 12.11 |
|  |  |  |  | *napA* | *napB* | *nirS* |  |  |  |  |
|  |  | Uncultured | *narG* | *narH* | *narl* | *nirK* | *norB* | *norC* | *nosZ* | 12.64 |
|  |  |  |  | *napA* | *napB* | *nirS* |  |  |  |  |
|  |  | *g__Gemmatimonas* | *narG* | *narH* | *narl* | *nirK* | *norB* | *norC* | *nosZ* | 8.87 |
|  |  |  |  | *napA* | *napB* | *nirS* |  |  |  |  |
|  |  | *g__Noviherbaspirillum* | *narG* | *narH* | *narl* | *nirK* | *norB* | *norC* | *nosZ* | 2.25 |
|  |  |  |  | *napA* | *napB* | *nirS* |  |  |  |  |
|  |  | *g__Klebsiella* | *narG* | *narH* | *narl* | *nirK* | *norB* | *norC* | *nosZ* | 1.35 |
|  |  |  |  | *napA* | *napB* | *nirS* |  |  |  |  |
|  |  | *g__Flavisolibacter* | *narG* | *narH* | *narl* | *nirK* | *norB* | *norC* | *nosZ* | 1.30 |
|  |  |  |  | *napA* | *napB* | *nirS* |  |  |  |  |
|  |  | *g__Rhodanobacter* | *narG* | *narH* | *narl* | *nirK* | *norB* | *norC* | *nosZ* | 1.29 |
|  |  |  |  | *napA* | *napB* | *nirS* |  |  |  |  |
|  | Total | |  |  |  |  |  |  |  | 39.81 |

**Table S4** The direct and indirect relationships between variables. The path coefficients are calculated by PLS-PM after 1000 bootstrap. SE: Soil type, FG: Gene abundance, BD: bacterial diversity, BCS: Bacterial community structure, N_2_O: nitrous oxide.

| ***nirK* gene containing bacteria** | | | |
| --- | --- | --- | --- |
| Relationships | Direct | Indirect | Total |
| SE -> FG | 0.408 | 0.000 | 0.408 |
| SE -> BD | 0.280 | 0.000 | 0.280 |
| SE -> BCS | 0.978 | 0.000 | 0.978 |
| SE -> N_2_O | 0.000 | 0.927 | 0.927 |
| FG -> BD | 0.000 | 0.000 | 0.000 |
| FG -> BCS | 0.000 | 0.000 | 0.000 |
| FG -> N_2_O | 0.133 | 0.000 | 0.133 |
| BD -> BCS | 0.000 | 0.000 | 0.000 |
| BD -> N_2_O | -0.149 | 0.000 | -0.149 |
| BCS -> N_2_O | 0.935 | 0.000 | 0.935 |
| ***nirS* gene containing bacteria** | | | |
| Relationships | Direct | Indirect | Total |
| SE -> FG | -0.062 | 0.000 | -0.062 |
| SE -> BD | -0.537 | 0.000 | -0.537 |
| SE -> BCS | -0.917 | 0.000 | -0.917 |
| SE -> N_2_O | 0.000 | 0.842 | 0.842 |
| FG -> BD | 0.000 | 0.000 | 0.000 |
| FG -> BCS | 0.000 | 0.000 | 0.000 |
| FG -> N_2_O | 0.310 | 0.000 | 0.310 |
| BD -> BCS | 0.000 | 0.000 | 0.000 |
| BD -> N_2_O | 0.040 | 0.000 | 0.040 |
| BCS -> N_2_O | -0.962 | 0.000 | -0.962 |
| ***norB* gene containing bacteria** | | | |
| Relationships | Direct | Indirect | Total |
| SE -> FG | -0.283 | 0.000 | -0.283 |
| SE -> BD | -0.626 | 0.000 | -0.626 |
| SE -> BCS | 0.994 | 0.000 | 0.994 |
| SE -> N_2_O | 0.000 | 0.971 | 0.971 |
| FG -> BD | 0.000 | 0.000 | 0.000 |
| FG -> BCS | 0.000 | 0.000 | 0.000 |
| FG -> N_2_O | 0.066 | 0.000 | 0.066 |
| BD -> BCS | 0.000 | 0.000 | 0.000 |
| BD -> N_2_O | -0.050 | 0.000 | -0.050 |
| BCS -> N_2_O | 0.963 | 0.000 | 0.963 |
| ***nosZ* gene containing bacteria** | | | |
| Relationships | Direct | Indirect | Total |
| SE -> FG | -0.512 | 0.000 | -0.512 |
| SE -> BD | -0.688 | 0.000 | -0.688 |
| SE -> BCS | -0.916 | 0.000 | -0.916 |
| SE -> N_2_O | 0.000 | 0.853 | 0.853 |
| FG -> BD | 0.000 | 0.000 | 0.000 |
| FG -> BCS | 0.000 | 0.000 | 0.000 |
| FG -> N_2_O | -0.104 | 0.000 | -0.104 |
| BD -> BCS | 0.000 | 0.000 | 0.000 |
| BD -> N_2_O | -0.0001 | 0.000 | -0.0001 |
| BCS -> N_2_O | -0.872 | 0.000 | -0.872 |

1. **Supplementary figures**


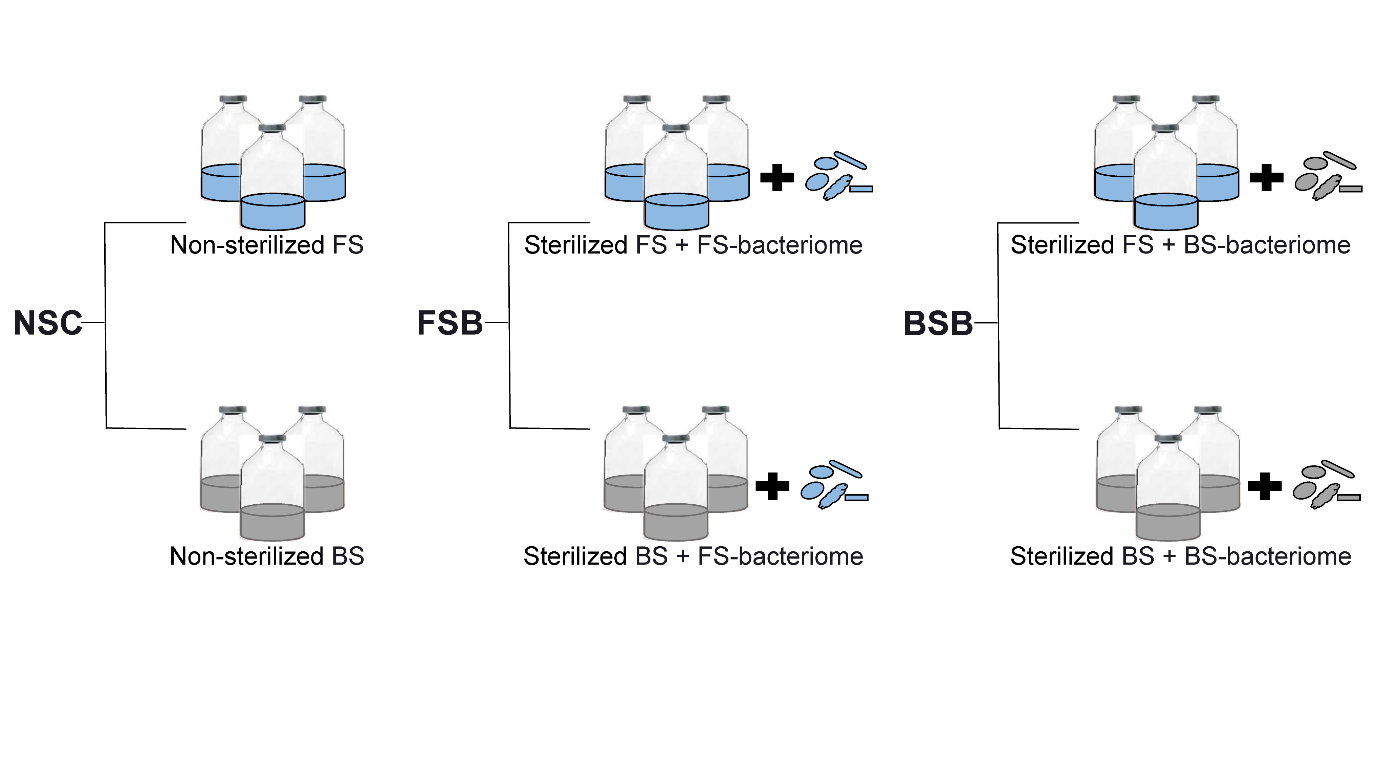


**Fig. S1** Experimental design


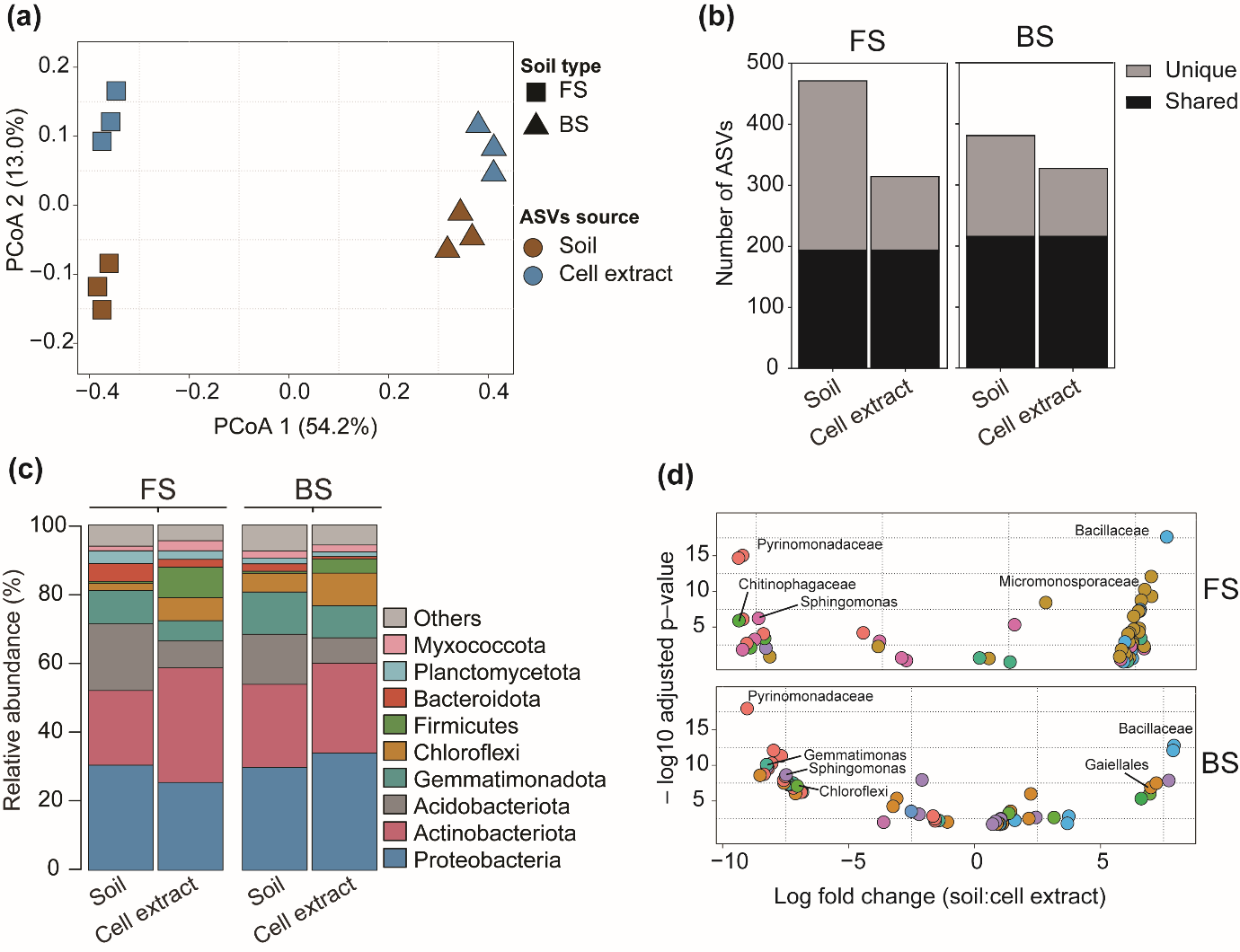


**Fig. S2** Extraction bias in bacterial communities caused by the cell extraction. PCoA plot based on Bray–Curtis distance shows community dissimilarity, where shapes represent origin soil and colours represent the source (soil or cell extract) **(a)**, zeta diversity depicts shared and unique ASVs between soil and cell extract sequences **(b)**, relative abundance differences at phylum level between soil and cell DNA extraction sources **(c)**, and fold changes in specific ASVs between soil and cell DNA extraction sources, calculated with an exact test **(d)**. ASVs with significant changes are labelled by taxon.

**
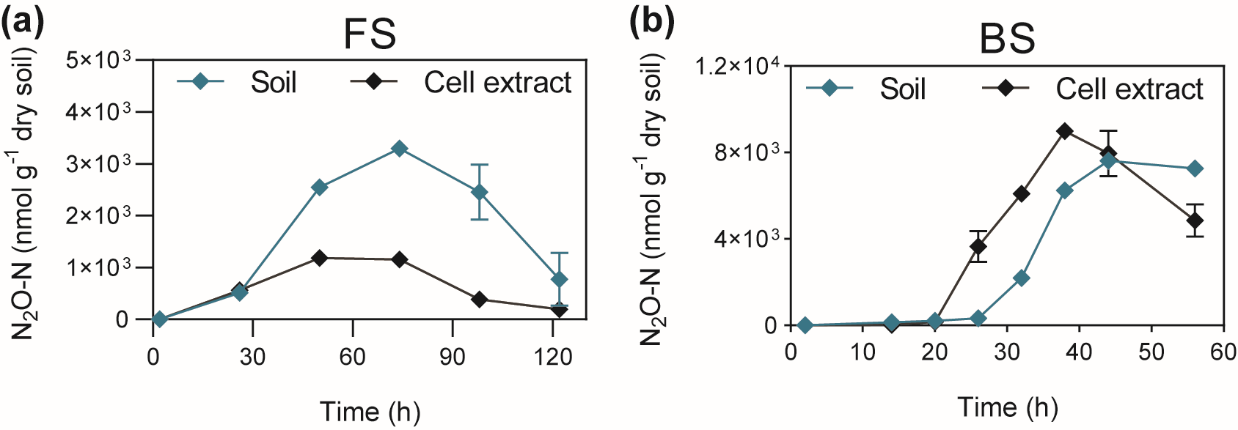
**

**Fig. S3** Kinetics of N_2_O emission between cell extract and original soil of FS **(a)** and BS **(b)**.


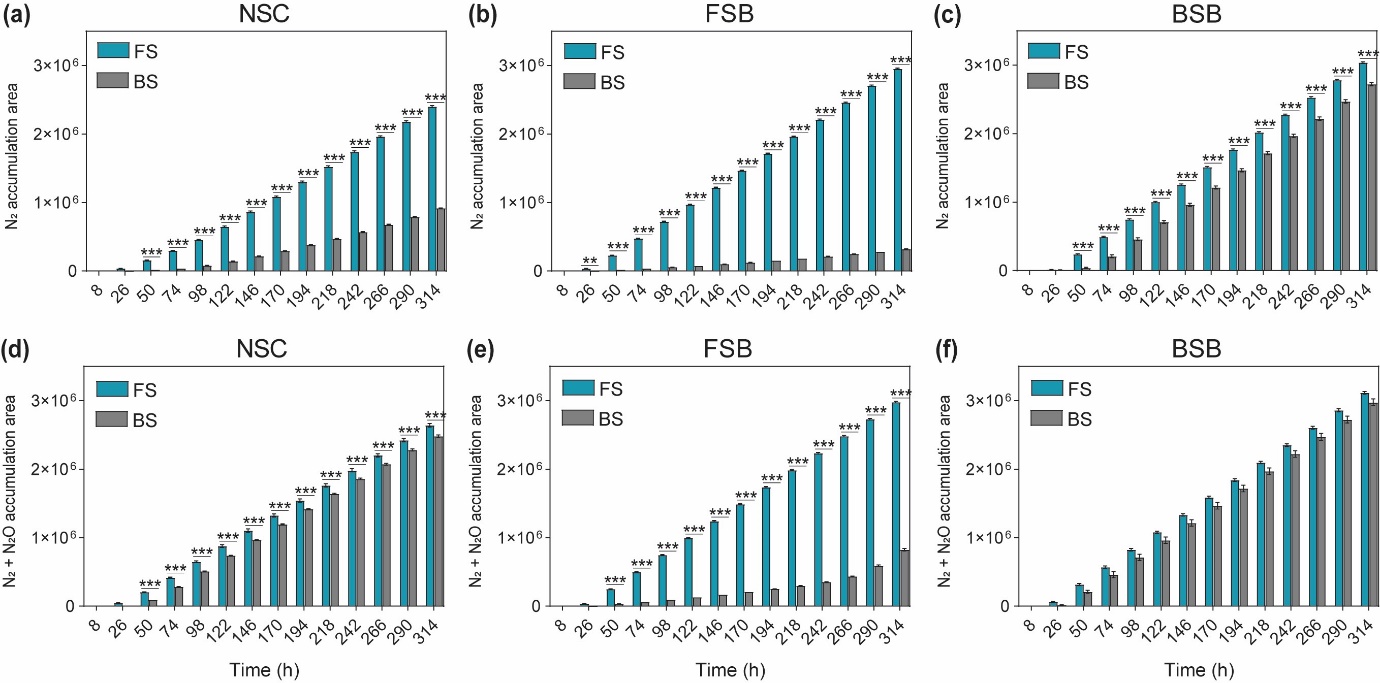


**Fig. S4** Comparison of the accumulation areas under the dynamic curve of N_2_ between FS and BS within treatment groups NSC **(a)**, FSB **(b)**, and BSB **(c)**, during anoxic incubation, and N_2_O+N_2_ accumulation area **(d–f)** in three different groups. Bars indicate means, and error bars indicate the standard error of mean (SEM). Differences in the peak areas of N_2_O and N_2_O+N_2_ were calculated via two-way ANOVA followed by Tukey’s multiple comparisons test and denoted by ** (p < 0.01) and *** (p < 0.001).


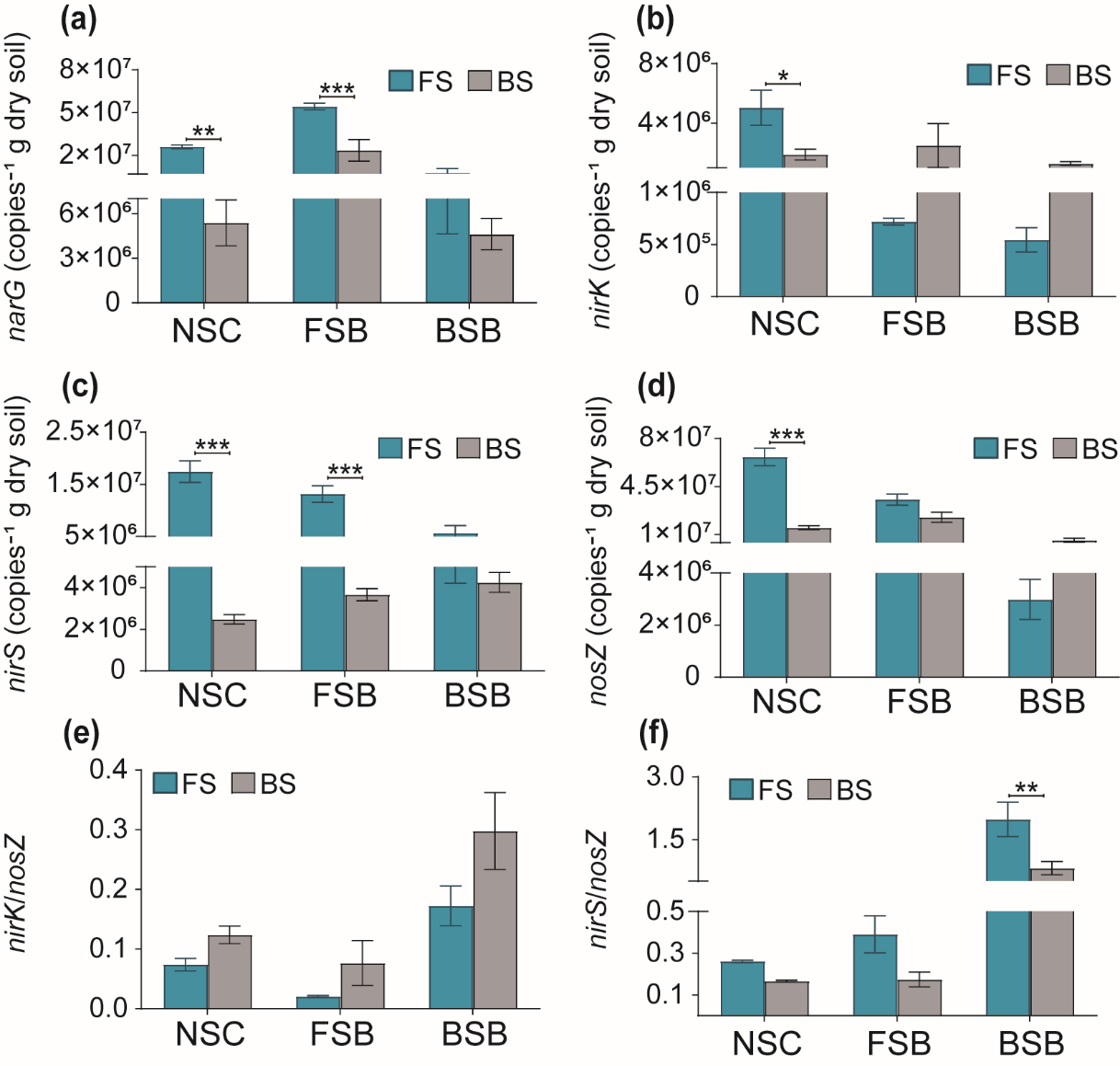


**Fig. S5** Quantity of denitrifying functional genes in different treatments. Differences in the quantity of narG **(a)**, nirK **(b)**, nirS **(c)**, and nosZ **(d)** genes among groups. Differences in the ratios of the nirK/nosZ **(e)** and nirS/nosZ **(f)** among treatment groups. Bars indicate means, and error bars indicate the SEM. Significant differences were calculated via two-way ANOVA and denoted by * (p < 0.05), ** (p < 0.01), and *** (p < 0.001).


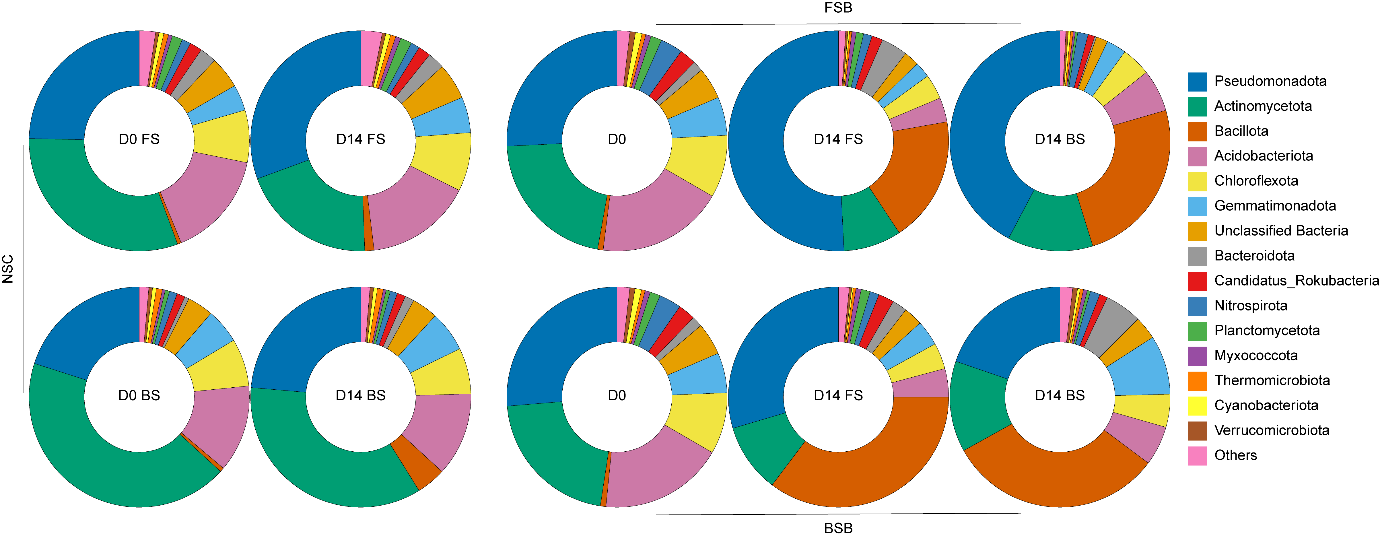


**Fig. S6** Variations in the relative abundance of dominant bacterial phyla in different treatment groups (NSC, FSB, and BSB) during anaerobic incubation are presented. The donut charts illustrate the composition of bacterial communities, with each color in the legend representing a different phylum.


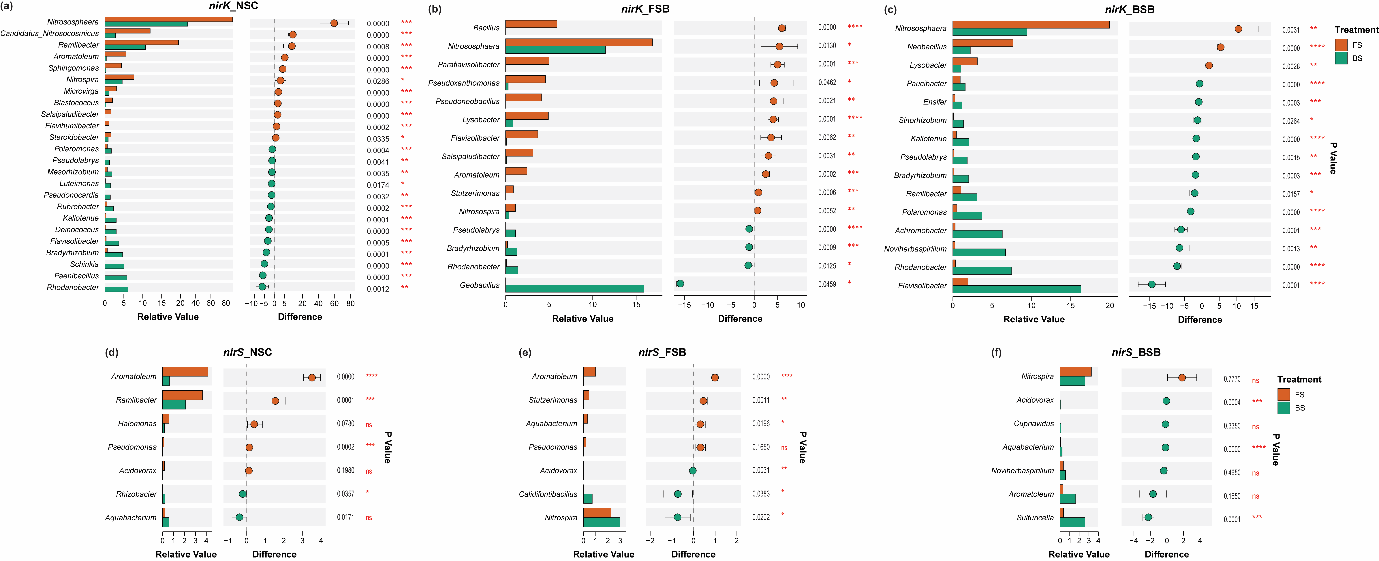
­

**Fig. S7** Differences in the relative abundance of denitrifying genera containing nirK and nirS genes across different treatment groups. Relative abundance of bacterial genera in FS and BS containing the nirK gene across NSC (a), FSB (b), and BSB (c) groups at Day 14. Relative abundance of bacterial genera in FS and BS containing the nirS gene across NSC (d), FSB (e), and BSB (f) groups at Day 14. The left panels display bar plots of the relative value of each genus, while the dot plots in the right panels show the confidence intervals (CI) for the differences in relative abundance between FS and BS. Significant differences are calculated via the Kruskal-Wallis test and denoted by * (p < 0.05), ** (p < 0.01), *** (p < 0.001), and **** (p < 0.0001).


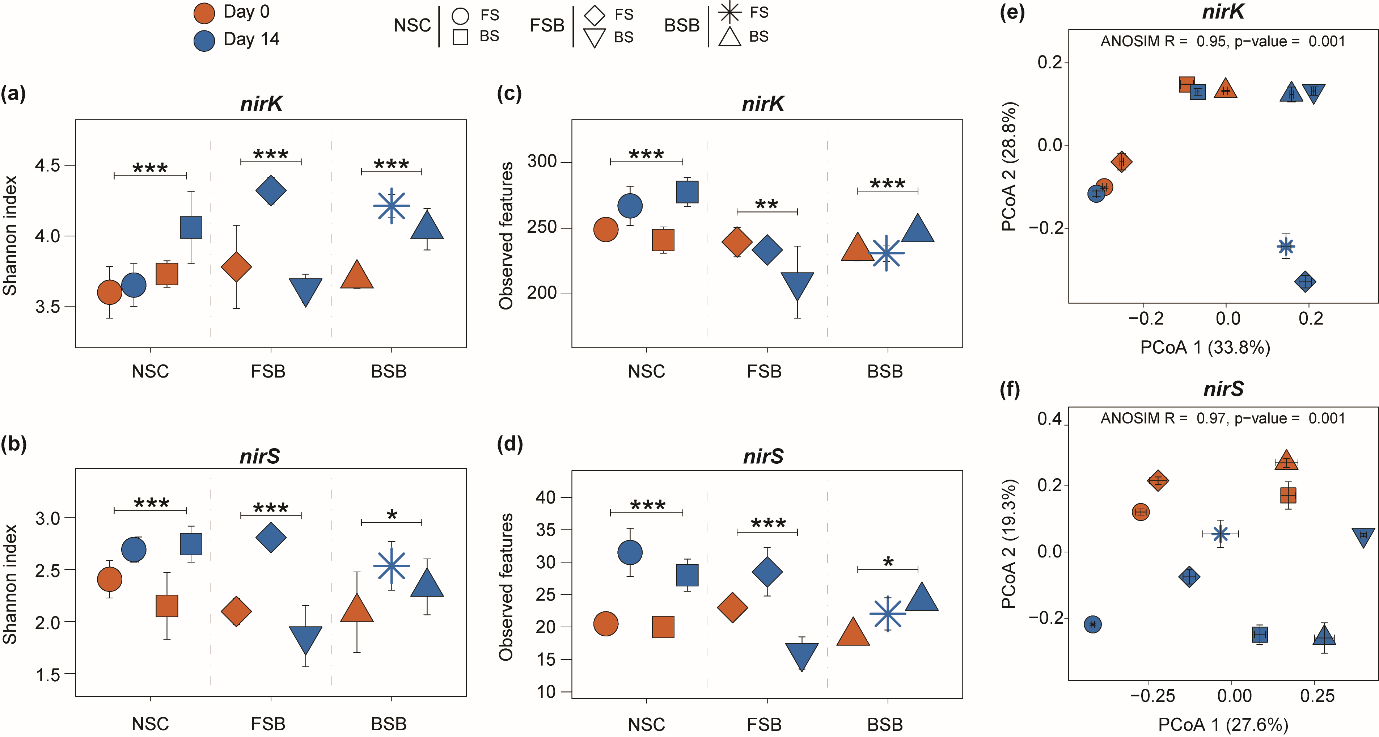


**Fig. S8** Composition and diversity of the nirK and nirS type denitrifying community in FS and BS across different treatment groups. Shannon index of the nirK denitrifying community **(a)** and nirS denitrifying community **(b)** at Day 0 and Day 14 across NSC, FSB, and BSB treatment groups. Observed features of the nirK denitrifying community **(c)** and nirS denitrifying community **(d)** at Day 0 and Day 14 across NSC, FSB, and BSB treatment groups. Significant differences are indicated by the Kruskal-Wallis test, denoted by * (p < 0.05), ** (p < 0.01), and *** (p < 0.001). PCoA based on Bray-Curtis dissimilarity showing the nirK denitrifying community **(e)** and nirS denitrifying community **(f)** structure at Day 0 and Day 14 across NSC, FSB, and BSB treatment groups. The p-value = 0.001 based on ANOSIM statistical test indicates significant differences.


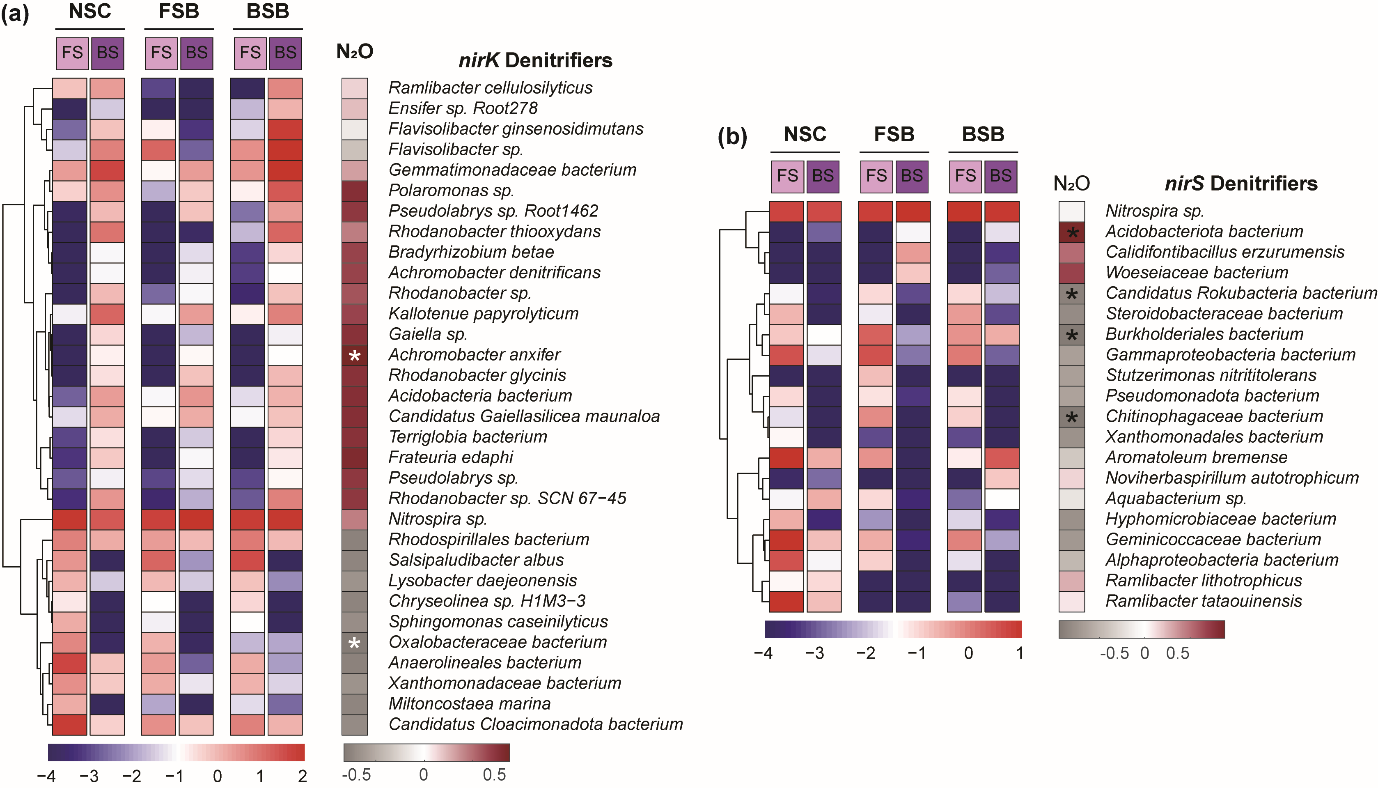


**Fig. S9** Correlation analysis of key denitrifiers with N_2_O accumulation in FS and BS across different treatment groups. Heatmap of LEfSe-derived key nirK denitrifiers **(a)** and nirS denitrifiers **(b)** identified for discriminating FS and BS across different treatment groups. The colors of the spots in the left panel represent the log-transformed relative abundance of the species in each sample, while those in the right panel denote the R-value of Spearman’s correlation between key species and N_2_O accumulation (* indicates p < 0.05).

**References**

1. Tarafder P, Rathore D: Spectrophotometric determination of nitrite in water. *Analyst* 1988, 113(7):1073-1076.

2. Govindasamy P, Mahawer SK, Mowrer J, Bagavathiannan M, Prasad M, Ramakrishnan S, Halli HM, Kumar S, Chandra A: Comparison of Low-Cost Methods for Soil Water Holding Capacity. *Communications in Soil Science and Plant Analysis* 2023, 54(2):287-296.

3. Wu Q, Ji M, Yu S, Li J, Wu X, Ju X, Liu B, Zhang X: Distinct Denitrifying Phenotypes of Predominant Bacteria Modulate Nitrous Oxide Metabolism in Two Typical Cropland Soils. *Microbial Ecology* 2022.

4. Henry S, Baudoin E, López-Gutiérrez JC, Martin-Laurent F, Brauman A, Philippot L: Quantification of denitrifying bacteria in soils by nirK gene targeted real-time PCR. *Journal of microbiological methods* 2004, 59(3):327-335.

5. Throbäck IN, Enwall K, Jarvis Å, Hallin S: Reassessing PCR primers targeting nirS, nirK and nosZ genes for community surveys of denitrifying bacteria with DGGE. *FEMS Microbiology Ecology* 2004, 49(3):401-417.

6. Bru D, Sarr A, Philippot L: Relative Abundances of Proteobacterial Membrane-Bound and Periplasmic Nitrate Reductases in Selected Environments. *Applied and Environmental Microbiology* 2007, 73(18):5971-5974.

7. Henry S, Bru D, Stres B, Hallet S, Philippot L: Quantitative detection of the nosZ gene, encoding nitrous oxide reductase, and comparison of the abundances of 16S rRNA, narG, nirK, and nosZ genes in soils. *Applied and environmental microbiology* 2006, 72(8):5181-5189.
